# Quantifying arm swing in Parkinson’s disease: a method accounting for arm activities during free-living gait

**DOI:** 10.1186/s12984-025-01578-z

**Published:** 2025-02-26

**Authors:** Erik Post, Twan van Laarhoven, Yordan P. Raykov, Max A. Little, Jorik Nonnekes, Tom M. Heskes, Bastiaan R. Bloem, Luc J. W. Evers

**Affiliations:** 1 Department of Neurology, Center of Expertise for Parkinson and Movement Disorders, Donders Institute for Brain, Cognition and Behaviour, Radboud University Medical Center, Nijmegen, The Netherlands; 2 Institute for Computing and Information Sciences, Radboud University, Nijmegen, The Netherlands; 3 University of Nottingham, Nottingham, UK; 4 University of Birmingham, Birmingham, UK

**Keywords:** Parkinson’s disease, Gait, Arm swing, Hypokinesia, Wearables, Wrist-worn sensors, Digital biomarkers

## Abstract

**Background:**

Accurately measuring hypokinetic arm swing during free-living gait in Parkinson’s disease (PD) is challenging due to other concurrent arm activities. We developed a method to isolate gait segments without these arm activities.

**Methods:**

Wrist accelerometer and gyroscope data were collected from 25 individuals with PD and 25 age-matched controls while performing unscripted activities in their home environment. This was done after overnight withdrawal of dopaminergic medication (‘pre-medication’) and approximately one hour after intake (‘post-medication’). Using video annotations as ground truth, we trained and evaluated two classifiers: one for detecting gait and one for detecting gait segments without other arm activities. Based on the filtered gait segments, arm swing was quantified using the median and 95th percentile range of motion (RoM). These arm swing parameters were evaluated in three ways: (1) the agreement between predicted and video-annotated gait segments without other arm activities, (2) the sensitivity to differences between PD and controls, and (3) the sensitivity to the effects of dopaminergic medication.

**Results:**

On the most affected side, the mean (SD) balanced accuracy for detecting gait without other arm activities was 0.84 (0.10) pre-medication and 0.88 (0.09) post-medication. The agreement between arm swing parameters of predicted and video-annotated gait segments without other arm activities was high irrespective of medication state (intra-class correlation coefficients: median RoM: 0.99; 95th percentile RoM: 0.97). Both the median and 95th percentile RoM were smaller in PD pre-medication compared to controls (median: $\Delta = -18.80^{\circ }$, 95% CI [$-$30.63, $-$10.60], *p* < 0.001; 95th percentile: $\Delta = -28.34^{\circ }$, 95% CI [$-$38.26, $-$18.18], *p* < 0.001), and smaller in pre- compared to post-medication (median: $\Delta = -12.31 ^{\circ }$, 95% CI [$-$21.35, $-$5.59], *p* < 0.001; 95th percentile: $\Delta = -19.04 ^{\circ }$, 95% CI [$-$28.48, $-$11.14], *p* < 0.001). The differences in RoM between pre- and post-medication were larger after filtering gait for the median (*p* < 0.01) and 95th percentile RoM (*p* = 0.01).

**Conclusions:**

Filtering out gait segments with other concurrent arm activities is feasible and increases the change in arm swing parameters following dopaminergic medication in free-living conditions. This approach may be used to monitor treatment effect and disease progression in daily life.

**Supplementary Information:**

The online version contains supplementary material available at 10.1186/s12984-025-01578-z.

## Introduction

A reduced arm swing during gait is a characteristic and early sign of Parkinson’s disease (PD) [1]. Initially, the reduction in arm swing is unilateral and stronger on the most affected side, while often progressing to a bilateral and more symmetrical presentation in later stages of the disease [2]. This reduction in arm swing is associated with both hypokinesia and rigidity [3, 4]. Arm swing in individuals with PD typically increases after dopaminergic medication intake [5]. As such, quantifying arm swing could provide insights into the response to medication. Also, because arm swing reduction is an early and progressive sign, quantifying long-term changes in arm swing over time could also serve to measure disease progression. This is particularly relevant in the context of clinical trials investigating disease-modifying therapies [6].

Currently, the assessment of arm swing reduction in individuals with PD relies on in-clinic observations. These observations are typically made by assessors while the individual with PD performs short walking tasks included in rating scales such as the Movement Disorder Society - Unified Parkinson’s Disease Rating Scale (MDS-UPDRS). However, the efficacy of in-clinic observations is hampered by the subjective nature of the examination—leading to observer bias [7], as well as inter- and intra-rater variability [8], the snapshot nature of measurements [9], the use of ordinal scales that are insensitive to small changes [10], and the requirement to travel to a clinic. This limits their use for monitoring response fluctuations in the context of individual patient care, and for evaluating new interventions such as disease modifying therapies in clinical trials [11]. Continuous data collection through wearable sensors could address these limitations [12–14].

Objectively quantifying arm swing in individuals with PD has gained increasing attention over the past decade, initially focusing on in-clinic assessments. For example, in-clinic motion-capture systems have shown reduced arm swing amplitude, velocity, and increased asymmetry in individuals with PD compared to age-matched healthy controls [5, 15–17]. Similar differences between PD and healthy controls have been observed in arm swing measures derived from wrist-worn sensor data collected in the clinic [18–23]. In addition, arm swing measures improved after dopaminergic medication intake [5, 24] and decreased over time [23]. Subtle changes in arm swing measures were observed during the prodromal phase in individuals with rapid eye movement sleep behavior disorder (RBD), and in non-manifesting carriers of the LRRK2-G2019S mutation [25, 26]. However, in-clinic assessments are often not representative of how individuals with PD function in daily life [23, 27]. These differences can be partly attributed to environmental factors, the effects of medication and stress, the awareness of being monitored, and task complexity [28–34].

When monitoring arm swing outside the clinic, it is important to distinguish between active and passive monitoring. Active monitoring involves standardized tests performed at home, while passive monitoring captures data during everyday activities. In active monitoring, arm swing parameters have been shown to be sensitive to dopaminergic medication intake [33]. Although active monitoring can provide valuable insights, it remains episodic, may be affected by a perceived awareness of being monitored, and relies on active participation from individuals, which can lead to declining retention rates in longitudinal studies [7, 35].

In contrast, passive monitoring offers continuous data collection in daily life without the drawbacks of active monitoring. Continuous data collected from wrist-worn sensors enable the detection and quantification of gait in free-living conditions [36–39]. Gait quality measures derived from these data have shown differences between individuals with PD and matched controls [37], and are also sensitive to the effects of dopaminergic medication [5, 36]. However, the heterogeneity of other arm activities conducted during free-living gait may interfere with obtaining accurate estimates of arm swing parameters when using wrist-worn sensors. Examples of these arm activities during gait include carrying an object, resting hands in trouser or jacket pockets, or making hand gestures (e.g., waving to a passing friend). To enable valid analysis of reduced arm swing in free-living conditions, methods are needed to detect and filter out such arm activities.

To this end, we aim to develop and validate a model for detecting other arm activities during gait. This would enable a more accurate estimation of arm swing parameters by focusing on gait segments free of other arm activities. Our approach involves an open-source analysis pipeline utilizing data from wrist-worn accelerometers and gyroscopes to (1) detect gait, (2) identify gait segments without other arm activities, and (3) quantify the arm swing range of motion (RoM) in the remaining segments. To evaluate our approach, we compare the predictions of steps (1) and (2) against ground truth video annotations from the Parkinson@Home Validation study [40]. To assess the added value of isolating gait segments without other arm activities, we determine whether the improvement in arm swing RoM after dopaminergic medication intake is more pronounced after filtering.

## Methods

**Fig. 1:**
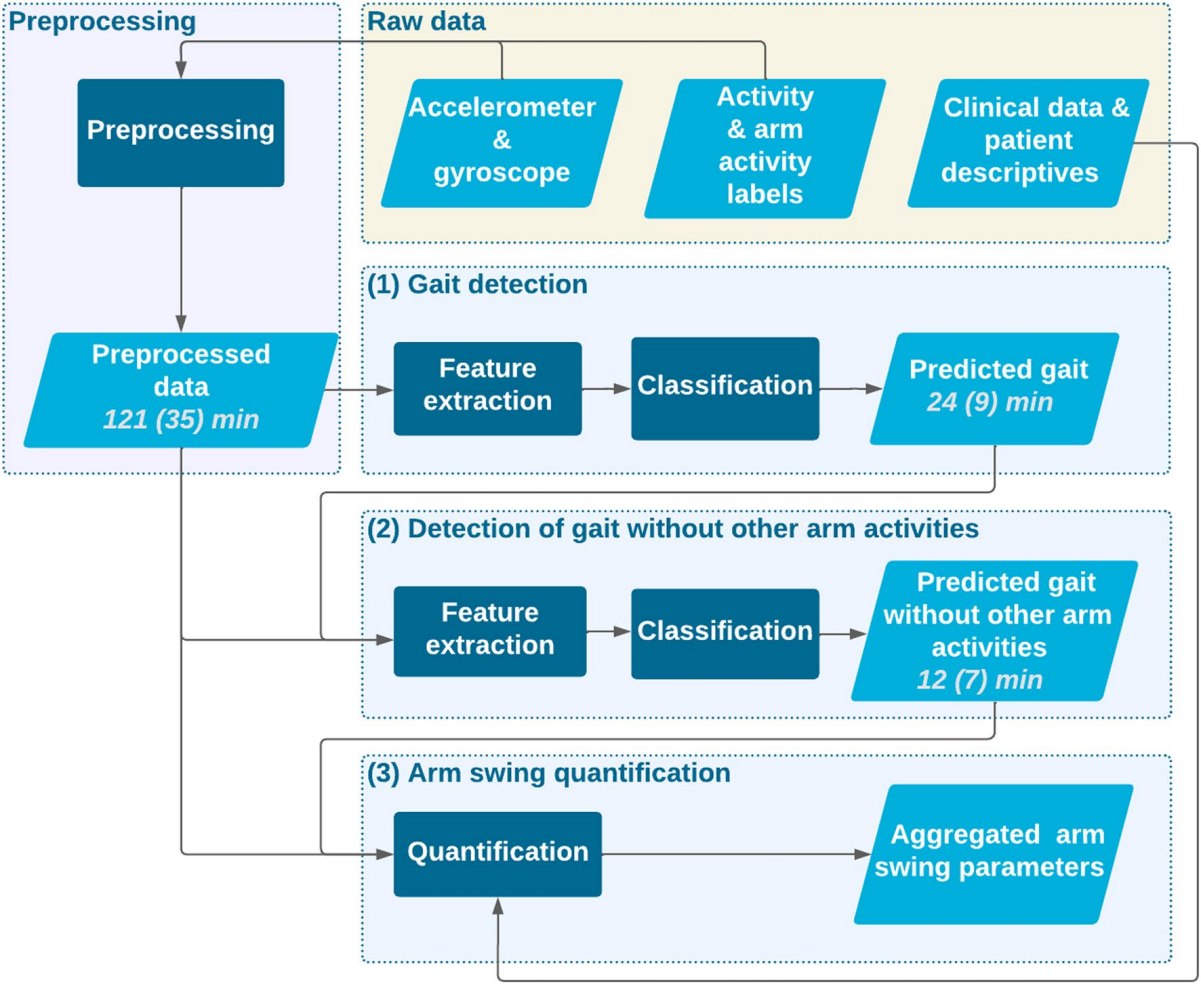
Data processing pipeline. The yellow area represents the input data, the pink area denotes preprocessing, and the blue areas highlight the three core components of the pipeline: (1) gait detection, (2) detection of gait without other arm activities, and (3) arm swing quantification. In the diagram, light blue parallelograms represent data, dark blue squares represent data processing methods, and transparent diamonds represent data selection processes. The numbers in the parallelograms representing data indicate the mean (SD) minutes of data remaining aggregated across all participants with PD

A high-level overview of the pipeline components is provided in Fig. 1. After raw data preprocessing, the pipeline consists of three consecutive steps: (1) gait detection, (2) detection of gait without other arm activities, and (3) arm swing quantification. We adopted a modular approach for gait detection and detecting other arm activities to allow easy replacement of individual components. The entire pipeline is publicly accessible and requires Python 3.11 (see section ’Availability of data and materials’).

### Dataset

The data were collected during the Parkinson@Home validation study, which included 25 individuals with PD and 25 age-matched controls [40]. This study collected wearable sensor data and video recordings during unscripted daily life activities in the participants’ home environment. Relevant summary statistics of the study sample can be found in Table 1. Characteristics of each participant with PD can be found in Table S1. For a detailed description we refer to the study documentation in the linked data repository and the original publication of the study [40, 41].

In brief, participants performed unscripted activities in and around their home for at least 1 h. Participants with PD did this twice: once in a practically defined OFF state (i.e., after an overnight withdrawal of dopaminergic medication, pre-med), and once one hour after taking their regular dopaminergic medication (post-med). This resulted in a mean (SD) of 150 (26) minutes of data for the PD group and 95 (17) minutes for controls. An assessor conducted MDS-UPDRS clinical assessments in both pre-med and post-med states prior to carrying out unscripted activities. Inclusion criteria for the PD group were: (1) a diagnosis of PD by a neurologist according to accepted international criteria, (2) use of levodopa and/or a dopamine agonist, (3) at least slight motor fluctuations (MDS-UPDRS Part IV item 4.3 $\ge$ 1), and (4) at least some Parkinson-related gait impairments (MDS-UPDRS Part II item 2.12 $\ge$ 1 and/or item 2.13 $\ge$ 1). For the current study, participants using a walking stick (n = 2) were excluded due to the lack of free arm swing on one side. We also excluded one individual with PD whose wrist-worn sensors shifted during data collection. Most participants with PD had mild to moderate disease severity (77% Hoehn & Yahr stages I and II), and no statistically significant differences in age and gender were found between the PD and control groups (age: *p* = 0.64; gender: *p* = 0.89). We computed an MDS-UPDRS Part III subscore composed of items most closely related to reduced arm swing, which included all lateral bradykinesia and rigidity items. A list of the included scores can be found in Table S4.

For this study, we used triaxial accelerometer and gyroscope data collected during unscripted activities both before and after medication intake. Both sensor modalities were sampled at 200 Hz using Gait Up Physilog 4 devices worn on both wrists of the participants. For the PD group, we present results separately for the most affected side (MAS) and least affected side (LAS), as determined by the unilateral MDS-UPDRS Part III pre-med subscore. In the case of equal lateral scores, the self-reported most affected side was used. When comparing PD with controls, participants were matched for self-reported hand dominance.

Video recordings of participants were made using a single hand-held video recording device with a sampling rate of 20 frames per second (fps) and a resolution of 640 (width) by 360 (height), operated by a second assessor. While recording, the participant was in view as much as possible. However, the participant’s movements between rooms resulted in a mean (SD) of 68 (42) seconds of missing recordings across participants. Furthermore, to limit the effect of participants’ awareness of being monitored on behavior, two assessors conducted the study visit: one recording the visit and one interacting with the participant. An exit survey showed that only 10% of participants felt uncomfortable being filmed in their own home. Synchronized video recordings of the unscripted activities were annotated by trained research assistants following a predefined protocol [40]. The annotations identified the presence of various daily life activities (including gait, sitting, standing, cycling and running) as well as tremor. Gait was defined as any episode in which the participant took five or more consecutive steps, excluding walking the stairs and turning. To distinguish from gait, a movement was annotated as turning when the feet rotated at least $90^{\circ }$ from the heel-off of the first step until the foot flat of the final step. The definitions of all activities can be found in the video annotation protocol in the data repository [41]. Walking the stairs and turning were excluded because they involve additional biomechanical and cognitive demands, introducing variability unrelated to hypokinesia, making it harder to isolate its specific effect on gait [42]. Furthermore, assessing steady-state gait provides insights into motor function during routine walking rather than specific maneuvers that may not be performed frequently. The prevalence of all annotated activities is shown in Table S2.

Subsequently, arm activities during gait were annotated using the available video recordings (by E. Post). Annotations were made separately for the left and right arms. Categories of arm activities were defined concurrently with the annotation process to allow flexibility in the scheme. The start and end of each arm activity were marked by its initiation and finalization. Arm activity categories and statistics are presented in Table S3.

**Table 1:** Characteristics of Parkinson’s disease (PD) participants and controls included in this study

Characteristics	Group	
	PD	Controls
N (men)	22 (11)	24 (11)
Age	63.7 ± 6.6	63.4 ± 10.1
Years since diagnosis	6.9 ± 3.5	N/A
Hoehn & Yahr stage I	1	N/A
Hoehn & Yahr stage II	16	N/A
Hoehn & Yahr stage III	4	N/A
Hoehn & Yahr stage IV	1	N/A
MDS-UPDRS Part I	11.6 ± 5.6	3.9 ± 4.2
MDS-UPDRS Part II	10.6 ± 4.8	0.5 ± 1.2
MDS-UPDRS Part III pre-med	41.7 ± 13.1	$8.9 \pm 6.9^{\dagger }$
MDS-UPDRS Part III post-med	27.7 ± 11.3	$8.9 \pm 6.9^{\dagger }$
MDS-UPDRS Part III pre-med MAS subscore	12.5 ± 3.8	$3.0 \pm 3.4^{\dagger }$
MDS-UPDRS Part III pre-med LAS subscore	9.6 ± 3.5	$3.9 \pm 3.0^{\dagger }$
MDS-UPDRS Part III post-med MAS subscore	8.6 ± 3.4	$3.0 \pm 3.4^{\dagger }$
MDS-UPDRS Part III post-med LAS subscore	7.5 ± 2.7	$3.9 \pm 3.0^{\dagger }$
MDS-UPDRS Part IV	6.1 ± 3.0	N/A

$\dagger$ Controls are assessed only once

Part III of the Movement Disorder Society-sponsored Unified Parkinson’s Disease Rating Scale (MDS-UPDRS) was split up into pre-medication (pre-med) and post-medication (post-med) states. The subscore includes lateral MDS-UPDRS Part III items most closely related to reduced arm swing, calculated separately for the most affected side (MAS) and least affected side (LAS). Values with ± indicate mean ± standard deviation. The Hoehn & Yahr assessment was conducted in the practically defined OFF state for all participants, except for one, where it was determined using video footage posterior to the visit

### Raw data preprocessing

The 200 Hz triaxial accelerometer and gyroscope signals were downsampled to 100 Hz to improve processing efficiency by decimating by a factor of 2. The wrist-worn sensor could be worn in two different orientations. Figure S1 shows the axis directions of the accelerometer and gyroscope after inverting the axes to account for these orientations. A 4th order Butterworth high-pass filter with a cutoff frequency of 0.2 Hz was applied to each axis of the accelerometer to separate the dynamic and gravitational components of the signal [43]. The extracted gravitational component was retained and used separately as a measure of wrist orientation. Sensor data with missing video annotations, due to an inability to reliably determine activities, were discarded [mean (SD) 2.5 (2.6) mins].

### Data processing pipeline

#### Gait detection

In this step, we included only the accelerometer sensor to facilitate comparison with existing gait detection algorithms. A sliding Hann window of 6 s with a 5-second overlap was applied to the time series data. The Fast Fourier transform (FFT) was then used to transform the windowed temporal signal into the spectral domain. From the windowed accelerometer signal, a total of 34 features were extracted (see Table 2).

For each axis, features extracted included the log-transformed power in specific frequency bands: below gait [0$-$0.7 Hz], gait [0.7$-$3.5 Hz], rest tremor [3.5–8 Hz] and above rest tremor [8–25 Hz]). We added frequency bands specific to tremor as we anticipated the rhythmic characteristic of rest tremor – similarly found in gait—to interfere with the classifier’s ability to correctly classify non-gait. The frequency range for gait was chosen to contain the fundamental frequency and at least the first harmonic of gait movements, while limiting potential overlap with the rest tremor frequency band [40]. The dominant frequency, defined as the frequency with the highest power between 0 and 25 Hz, was also extracted for each axis.

From the gravitational component of each axis, we extracted the mean and standard deviation. The gravitational component represents the orientation of the wrist, which is expected to vary considerably between activities. Using the norm of the three axes of the dynamic component, we extracted the standard deviation in the temporal domain and Mel Frequency Cepstral Coefficients (MFCCs) in the spectral domain as measures of variability and rhythmicity. MFCCs are traditionally effective in audio analysis, but the rhythmic and repetitive nature of gait similarly enables MFCCs to be beneficial in the field of human activity recognition [44]. The MFCC parameters were set to capture information related to the harmonics of gait. A total of 12 MFCCs were extracted between 0 and 25 Hz using 15 filter banks.

**Table 2:** Features used in detecting gait

Feature	Axis	Sensor	# Features
Standard deviation temporal signal	Norm	Accelerometer	1
Mel Frequency Cepstral Coefficients	Norm	Accelerometer	12
Dominant frequency	Per axis	Accelerometer	3
Power in frequency bands	Per axis	Accelerometer	12
Gravitational component acceleration	Per axis	Accelerometer	6

Based on the video annotations, each window was labeled as either gait or non-gait using majority voting over the timestamps spanning the window. A complete list of activities and their prevalence can be found in Table S2. Least Absolute Shrinkage and Selector Operator (LASSO) regularized logistic regression (LR) and random forest (RF) were used as classification models for gait detection. The selection of these models was made to include both a relatively simple model (LR) and one capable of capturing non-linear relationships (RF). More advanced, data-intensive methods, such as deep learning, were omitted due to the limited size of the data in this study. We used nested leave-one-subject-out cross-validation (LOSO-CV) with grid search to find an optimal set of hyperparameters that maximized balanced accuracy in the inner loop (hyperparameters for LR: regularization parameter and number of iterations; hyperparameters for RF: number of estimators and maximum depth). Within each fold, the PD sample of the training data was used to set the classification threshold, ensuring a minimum specificity of 0.95 on the training set. This approach was adopted to limit the number of false-positive predictions, which were expected to interfere with the detection of gait without other arm activities. For logistic regression, features were standardized within each fold to avoid data leakage.

A single classifier was selected based on the highest balanced accuracy. For this classifier, we estimated the influence of gait segment duration and specific arm activities on its sensitivity. Additionally, we assessed the classifier’s robustness in detecting impaired gait by comparing sensitivity between PD and controls, between pre-med and post-med, between the most and least affected sides, and across varying MDS-UPDRS Part III pre-med subscores. To account for differences in behavior, we adjusted these sensitivities for the prevalence of gait segment durations in all group comparisons, and for the prevalence of other arm activities in all group comparisons except PD versus controls. The adjusted prevalence was determined at the group level.

#### Filtering gait: detection of gait without other arm activities

Using the predicted gait segments from step 1, the next step was to develop a model to detect gait segments without other arm activities. Examples of other arm activities include, but are not limited to, holding an object, gesturing, and opening a door. A complete list of arm activities and their prevalence can be found in Table S3. We refer to the predicted gait segments from step 1 as unfiltered gait, and the subset of these segments predicted to have no other arm activities as filtered gait.

Using only the predicted gait segments, we applied a sliding Hann window of 3 s with 75% overlap to the time series data, followed by a Fast Fourier transform (FFT) to convert the windows into the spectral domain. We included accelerometer features similar to those used in the gait detection step, as well as MFCCs from the gyroscope signal (see Table 3 for a complete list of features).

Based on the video annotations, each window was labeled as “gait with other arm activities” or “gait without other arm activities” using majority voting. Similar to the gait detection task, we evaluated a LR and RF classifier using nested LOSO-CV grid search. However, different from gait detection, the classification threshold for filtering gait was determined by optimizing for balanced accuracy on the training set across the PD cohort. This decision was made because false positives and false negatives were equally undesirable in this step.

We selected a single classifier based on the highest balanced accuracy and conducted an in-depth evaluation of its performance. This included analyzing the classifier’s specificity for detecting specific other arm activities and assessing the effect of gait segment duration on its sensitivity. Additionally, we evaluated the impact of PD on the classifier’s sensitivity. Given that severely hypokinetic arm swing during gait may resemble gait with certain other arm activities, such as keeping a hand in a pocket, we anticipated that this similarity could introduce bias by incorrectly filtering out gait segments with a strongly reduced arm swing. To investigate this potential effect, we compared the classifier’s sensitivity between pre-med and post-med, between the most and least affected sides, and across varying MDS-UPDRS Part III pre-med subscores. Comparisons between pre-med and post-med, and between the most and least affected sides, were adjusted for differences in gait segment duration, following the same approach used in the gait detection step (step 1).

**Table 3:** Features used in filtering gait

Feature	Axis	Sensor	# Features
Standard deviation temporal signal	Norm	Accelerometer	1
Mel Frequency Cepstral Coefficients	Norm	Accelerometer	12
Dominant frequency	Per axis	Accelerometer	3
Power in frequency bands	Per axis	Accelerometer	12
Gravitational acceleration	Per axis	Accelerometer	6
Mel Frequency Cepstral Coefficients	Norm	Gyroscope	12

#### Arm swing quantification

The filtered gait segments from step 3 were used to quantify the arm swing RoM in degrees ($^{\circ }$). We excluded participants from the PD group who had less than one minute of filtered gait in either the pre-med or post-med state, and participants from the control group who had less than one minute of filtered gait in the entire visit.

**Fig. 2:**
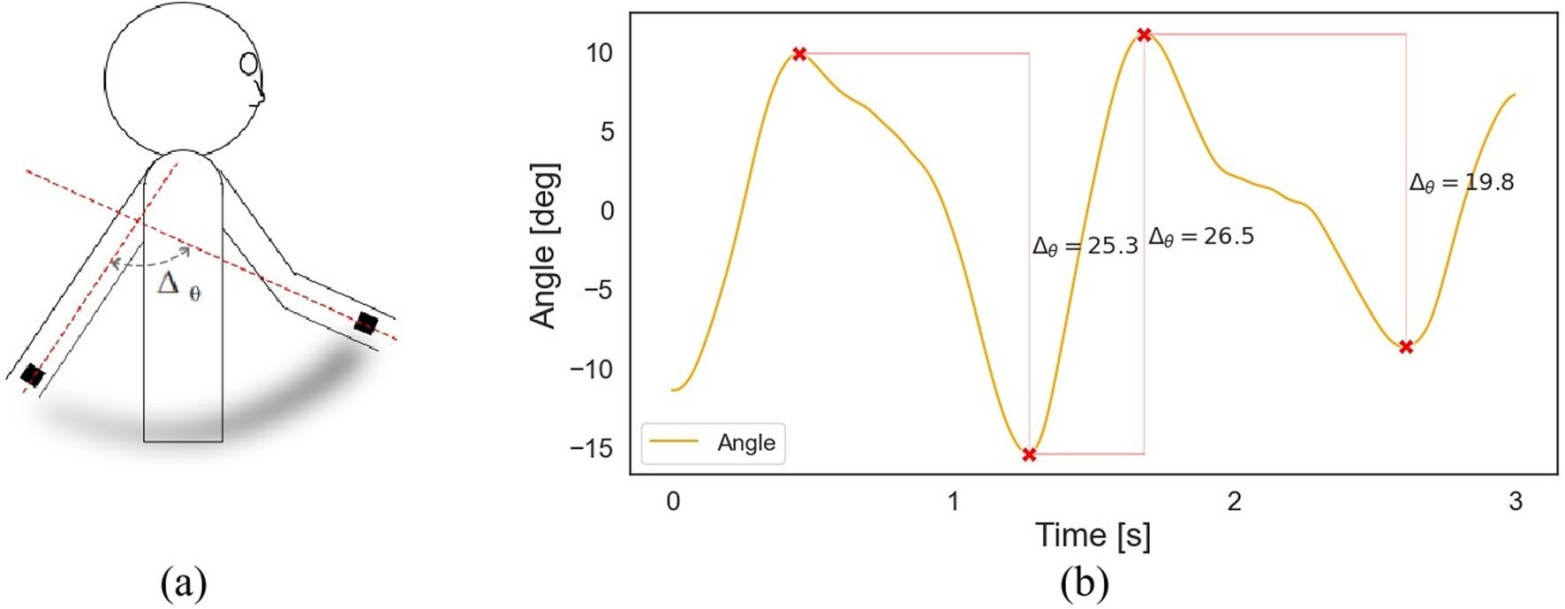
Illustration of the computation of the range of motion. **a** The motion of a single arm from a backward position to a forward position. The range of motion is defined as the difference, $\Delta _{\theta }$, in the estimated angle $\theta$ between the final points of consecutive forward and backward swings of the same arm. **b** An example of the estimated angle in degrees (deg) of a single time window, with detected peaks marked in red. The range of motion is calculated as the absolute difference between consecutive peaks

The RoM was computed following the methodology developed by Warmerdam and colleagues [18]. First, principal component analysis (PCA) was performed on the y-axis and z-axis of the gyroscope signals over all filtered gait segments—these two axes were expected to reflect arm swing most (see Figure S1 for axis directions). The first principal component was extracted to isolate the angular velocity in the direction of arm swing. Next, the principal component was numerically integrated to estimate the angle. To account for signal drift, a moving average was subtracted from the angle using a rolling window of one second. Peaks in the estimated angle were then automatically selected based on the following criteria: (1) a minimum could not be succeeded by another minimum, nor could a maximum be succeeded by another maximum; in cases of successive minima or maxima, the peak with the highest absolute value was selected, and (2) the time between peaks had to be at least 1/1.8 s; in cases of peaks occurring too close together, the last peak was discarded. The threshold of 1.8 s aligns with the selected frequency band for gait, where the first harmonic is not expected to exceed 1.8 Hz. Finally, the RoM was computed as the absolute differences between consecutive peaks (see Fig. 2).

We computed aggregations of the arm swing RoM per participant as candidate digital biomarkers (for the PD group: separately for the pre-med and post-med states). Specifically, we extracted the median RoM as a measure of general performance and the 95th percentile RoM as a measure of capacity.

To further validate the filtering step (step 2), we assessed whether the filter introduced bias by erroneously filtering out gait segments with a small arm swing RoM. This was evaluated by comparing the median and 95th percentile RoM for predicted versus video-annotated gait segments without other arm activities using Bland-Altman plots and intra-class correlation coefficients (ICCs).

Finally, we assessed the effect of filtering gait on the ability to measure changes in arm swing RoM following dopaminergic medication intake. First, we evaluated whether the arm swing parameters reflected expected differences between the PD and control groups, between pre-med and post-med states, and between the most and least affected sides. Next, we assessed the added value of filtering gait by comparing changes in arm swing parameters following dopaminergic medication intake between unfiltered and filtered gait. The change in the arm swing parameters following dopaminergic medication intake was defined for each participant as the difference between pre-med and post-med values.

### Model evaluation and statistical analysis

Model performance for gait detection (step 1) and filtering gait (step 2) was evaluated at the timestamp level. For each timestamp, predictions were determined using uniform majority voting over overlapping windows. Results for each step were stratified by gait segment duration. A gait segment was defined as a sequence of consecutive timestamps labeled as gait in the video annotations. A new segment was initiated if the gap between consecutive gait timestamps exceeded 1.5 s. Gait segments were classified as short (< 5 s), moderately long (5–10 s), long (10–20 s) or very long ($\ge$ 20 s).

Due to the non-normality of performance metrics and arm swing parameters across participants, statistical significance was assessed using the non-parametric Wilcoxon signed-rank test for dependent samples and the rank-sums test for independent samples. Statistical significance was denoted as follows: * (*p* < 0.05), ** (*p* < 0.01), *** (*p* < 0.001), and **** (*p* < 0.001). Effect sizes for post-hoc comparisons were reported as the median difference ($\Delta$) with a 95% confidence interval (CI). Correlations were assessed using Spearman’s rank-order correlation, denoted by $r_{s}$. All statistical analyses were performed in RStudio 2023.12.0.

## Results

### Gait detection

The amounts of video-annotated gait and non-gait data used in the following analyses are presented in Table 4.

**Table 4:** The duration (in minutes) of video-annotated gait and non-gait used

	Gait					Non-gait
	Short	Moderately long	Long	Very long		
	($< 5$ s)	($5{-}10$ s)	($10{-}20$ s)	($\ge 20$ s)	Total	Total
PD pre-med	0.9 (0.5)	1.8 (0.8)	1.9 (0.8)	9.3 (5.8)	13.8 (5.9)	77.9 (15.5)
PD post-med	0.5 (0.3)	1.3 (0.5)	1.1 (0.7)	8.8 (5.4)	11.6 (5.7)	45.5 (16.5)
Controls	1.7 (0.6)	2.3 (0.8)	1.8 (1.4)	14.3 (5.5)	20.1 (6.6)	75.4 (15.6)

The mean (SD) was calculated over all individuals constituting the gait segment duration stratification. PD is stratified by medication state: prior to (pre-med) and after medication intake (post-med)

#### Selecting a classifier for gait detection

The classification performance of the LR and RF classifiers for gait detection is shown in Table 5. Compared to the LR classifier, the RF classifier achieved higher balanced accuracy on the most affected side in both pre-med ($\Delta$ = 0.02, 95% CI [0.01, 0.03], *p* < 0.001) and post-med ($\Delta$ = 0.02, 95% CI [0.00, 0.03], *p* < 0.01). As a result, the RF classifier was selected for subsequent analyses. The feature coefficients of the LR classifier are shown in Figure S2, while the feature impurity scores of the RF classifier are shown in Figure S3.

**Table 5:** Performance of the classifiers for gait detection

Group	Sensitivity		Specificity		AUC	
	LR	RF	LR	RF	LR	RF
PD pre-med	0.83 (0.10)	0.89 (0.09)	0.96 (0.03)	0.95 (0.03)	0.98 (0.02)	0.98 (0.01)
PD post-med	0.88 (0.09)	0.95 (0.05)	0.94 (0.04)	0.91 (0.05)	0.98 (0.01)	0.99 (0.01)
Controls	0.92 (0.03)	0.96 (0.02)	0.88 (0.06)	0.82 (0.09)	0.97 (0.01)	0.98 (0.01)

Classifiers include the logistic regression (LR) and random forest (RF). Performance is stratified by pre-medication (pre-med) and post-medication (post-med) states in the PD group. Results are shown for the most affected side of PD participants, and for controls, matching of affected side stratifications based on self-reported hand dominance was applied. Performance metrics are presented as mean (SD) across participants

#### Effect of behavior on gait detection

We evaluated the effect of behavior on model performance by examining specificity for different non-gait activities and sensitivity to detecting gait across different arm activities and varying gait segment durations.

Non-gait activities that were frequently misclassified as gait primarily included walking the stairs, turning, and postural transitions (see Figure S4). Gait misclassified as non-gait was often attributed to arm activities such as grabbing, pulling, and pushing movements. In contrast, gait without other arm activities was particularly often correctly classified as gait (see Figure S5).

The classifier’s sensitivity increased with the duration of gait segments. The mean (SD) sensitivity in PD pre-med for short gait segments (< 5 s) was 0.63 (0.18), for moderately long gait segments (5–10 s) was 0.82 (0.13), for long gait segments (10–20 s) was 0.77 (0.17), and for very long gait segments (> 20 s) was 0.95 (0.13) (see Figure S6). Sensitivity for very long gait segments was significantly higher than for other gait segment durations: short ($\Delta$ = 0.32, 95% CI [0.24, 0.41], *p* < 0.001), moderately long ($\Delta$ = 0.12, 95% CI [0.08, 0.18], *p* < 0.001), and long ($\Delta$ = 0.17, 95% CI [0.08, 0.28], *p* < 0.001).

#### Effect of PD motor signs on gait detection

To assess whether PD and the severity of motor signs affected the ability to detect gait, we compared the sensitivity of the RF classifier across several groups: PD versus controls, pre-med versus post-med, the most affected versus the least affected sides (pre-med), and varying MDS-UPDRS Part III pre-med subscores.

Gait detection sensitivity was comparable between pre-med and post-med conditions ($\Delta$ = 0.00, 95% CI [$-$0.04, 0.08], *p* = 0.95), after correcting for differences in gait segment duration and arm activities. Sensitivity in controls was higher compared to pre-med ($\Delta$ = 0.06, 95% CI [0.03, 0.09], *p* < 0.001), but not higher compared to post-med ($\Delta$ = 0.00, 95% CI [$-$0.01, 0.02], *p* = 0.87). A more detailed analysis can be found in Figure S7.

Individuals with higher MDS-UPDRS Part III pre-med subscores did not show lower sensitivity pre-med for any gait segment duration, as shown in Figure S8. No significant differences were found between the most and least affected sides ($\Delta$ = 0.00, 95% CI [$-$0.02, 0.03], *p* = 0.68).

Lastly, we observed that tremor was rarely misclassified as gait, despite its periodic characteristics. The mean (SD) specificity of the classifier during tremor was 0.94 (0.06), which was not significantly different from the specificity of 0.93 (0.04) in the absence of tremor (*p* = 0.74).

### Filtering gait: detection of gait without other arm activities

The steps below were conducted by processing gait segments detected by the gait detection model (see Table 6 for the amount of detected gait). Note that only the PD group was used for training these classifiers because there were no arm activity video labels for the control group.

**Table 6:** Duration (in minutes) of predicted gait used as input for the following analyses

	Short	Moderately long	Long	Very long	Non-gait	Total
	($< 5$ s)	($5\text {-}{-}10$ s)	($10\text {-}{-}20$ s)	($\ge 20$ s)		
PD pre-med	0.6 (0.4)	1.5 (0.8)	1.5 (0.7)	8.9 (5.7)	4.1 (2.4)	16.5 (6.7)
PD post-med	0.4 (0.3)	1.1 (0.5)	1.0 (0.6)	8.7 (5.5)	3.8 (2.7)	14.9 (7.3)
Controls	1.4 (0.5)	2.1 (0.7)	1.6 (1.3)	14.2 (5.5)	12.7 (6.5)	32.5 (10.4)

The mean (SD) was calculated over all individuals constituting the gait segment duration stratification. Gait segment duration was determined based on annotated gait, and the ’non-gait’ category refers to annotated non-gait predicted as gait. PD participants are stratified by medication state: prior to (pre-med) and after medication intake (post-med). The duration shown was for the most affected side only, as results for the least affected side were comparable

#### Selecting a classifier for filtering gait

Classification performance of the LR and RF classifiers for detecting gait without other arm activities can be found in Table 7. We found no statistically significant difference in balanced accuracy between the classifiers ($\Delta$ = 0.00, 95% CI [$-$0.01, 0.03], *p* = 0.56). Therefore, we selected the LR classifier for its inherent simplicity. Feature coefficients of the LR classifier are shown in Figure S9, and feature impurity scores of the RF classifier are shown in Figure S10.

**Table 7:** Performance of the classifiers for detecting gait without other arm activities

Group	Sensitivity		Specificity		AUC	
	LR	RF	LR	RF	LR	RF
Pre-med MAS	0.75 (0.22)	0.72 (0.28)	0.92 (0.04)	0.93 (0.04)	0.93 (0.06)	0.92 (0.10)
Post-med MAS	0.85 (0.19)	0.86 (0.14)	0.90 (0.08)	0.89 (0.09)	0.96 (0.02)	0.96 (0.03)
Pre-med LAS	0.79 (0.17)	0.77 (0.20)	0.91 (0.06)	0.91 (0.10)	0.94 (0.04)	0.92 (0.09)
Post-med LAS	0.80 (0.23)	0.77 (0.28)	0.85 (0.14)	0.84 (0.17)	0.93 (0.08)	0.90 (0.14)

Classifiers include the logistic regression (LR) and the random forest (RF). The PD group is stratified by medication state (prior to (pre-med) and after medication intake (post-med)) and affected side (most affected side (MAS) and least affected side (LAS)). Performance metrics are presented as mean (SD) across participants

#### Effect of behavior on filtering gait

To better understand how behavior affects the classifier, we examined the effect of specific arm activities on specificity and the effect of gait segment duration on sensitivity.

**Fig. 3:**
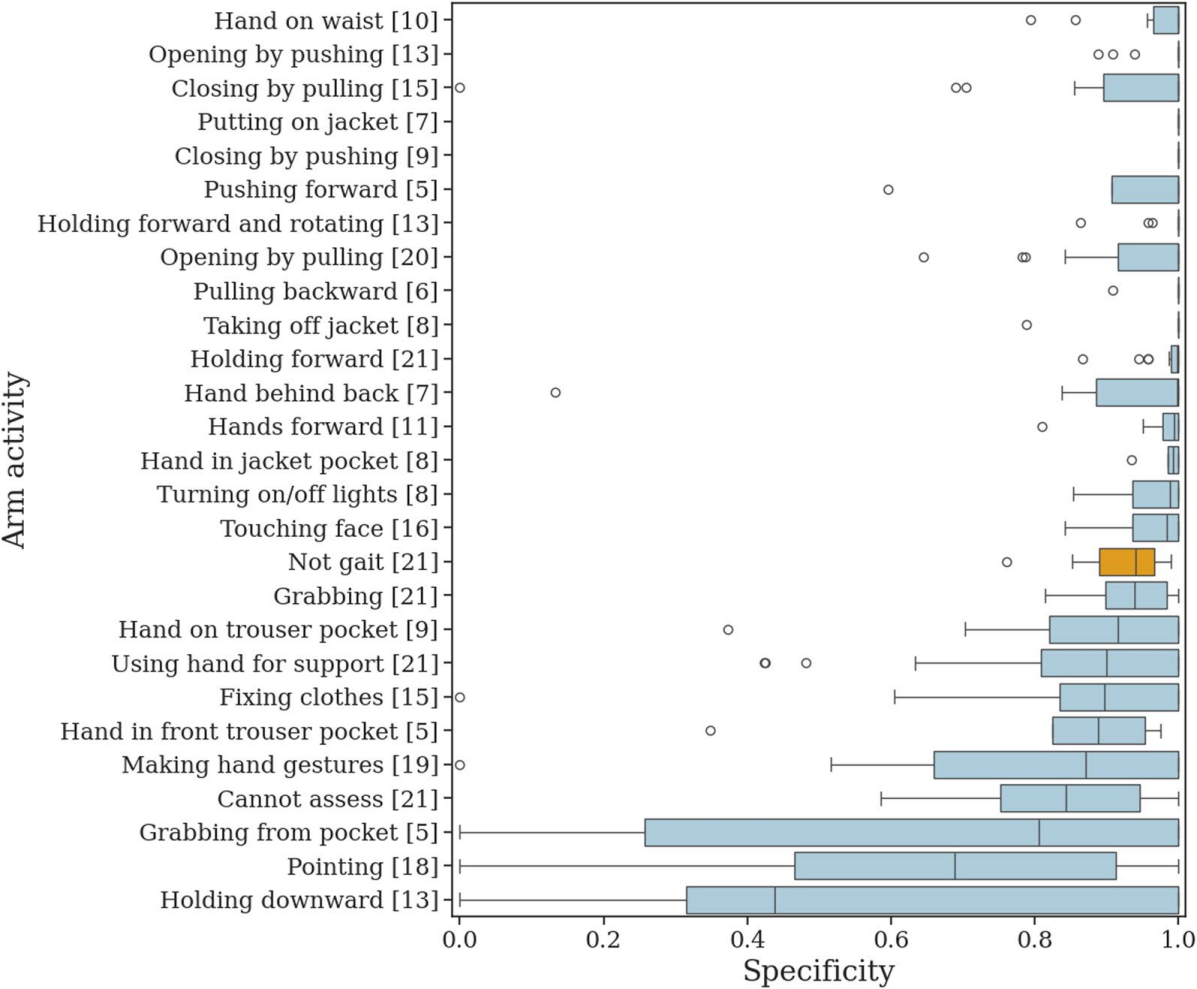
Specificity of the classifier detecting gait without other arm activities, stratified by arm activity. Since filtering gait was performed on predicted gait segments, the input included non-gait activities misclassified as gait, (e.g., turning or sitting), categorized as *Not gait* and highlighted in orange. The number in square brackets indicates the total number of participants exhibiting each arm activity

Fig. 3 displays the classifier’s specificity for each arm activity, showing considerable variability both within and between categories of arm activities. Arm activities with relatively low specificity were typically shorter in duration, such as making hand gestures, pointing, or grabbing an object. However, some arm activities of longer duration also exhibited low specificity, including holding an object downward, holding a dog leash, or keeping hands on front trouser pockets. In contrast, arm activities with consistently high specificity included actions such as opening and closing doors and cabinets, as well as holding objects forward.

The classifier’s sensitivity was influenced by the duration of gait segments (see Figure S11). For the most affected side pre-med, the mean (SD) sensitivity was 0.53 (0.14) in short segments, 0.70 (0.14) in moderately long segments, 0.83 (0.19) in long segments, and 0.88 (0.23) in very long segments.

#### Effect of PD motor signs on filtering gait

To investigate the potential effect of motor sign severity on the ability to correctly classify gait without other arm activities, we compared the sensitivity of the classifier between pre-med and post-med conditions (on the most affected side). Additionally, for pre-med, we compared the most affected side with the least affected side (see Table 7). The sensitivity was not higher in post-med compared to pre-med after adjusting for differences in gait segment duration ($\Delta = 0.02^{\circ }$, 95% CI [$-$0.04, 0.21], *p* = 0.27). We also did not find a lower sensitivity in pre-med on the most affected side compared to the least affected side ($\Delta = 0.01^{\circ }$, 95% CI [$-$0.04, 0.05], *p* = 0.86).

To further validate that hypokinesia severity did not affect the ability to detect gait without other arm activities, we examined the correlation between the sensitivity in pre-med most affected side and the MDS-UPDRS Part III pre-med subscore across gait segment duration categories (see Figure S13). Individuals with a higher MDS-UPDRS Part III pre-med subscore showed a lower sensitivity in moderately long segments ($r_{s}$ = $-$0.62, 95% CI [$-$0.83, $-$0.26], *p* < 0.01), long segments ($r_{s}$ = $-$0.67, 95% CI [$-$0.87, $-$0.30], *p* < 0.01), and in very long segments ($r_{s}$ = $-$0.51, 95% CI [$-$0.81, $-$0.03], *p* = 0.04). We did not observe this correlation in short segments ($r_{s}$ = $-$0.34, 95% CI [$-$0.67, 0.11], *p* = 0.13).

### Arm swing quantification

The following analyses were conducted using the filtered gait segments. The amount of gait data remaining after filtering out gait segments with other arm activities is shown in Table 8. Two PD participants with less than one minute of filtered gait in either the pre-med or post-med state were excluded.

**Table 8:** Amount of filtered gait in minutes stratified by gait segment duration

Group	Short	Moderately long	Long	Very long		
	($< 5$ s)	($5{-}10$ s)	($10{-}20$ s)	($\ge 20$ s)	Non-gait	Total
PD pre-med	0.3 (0.3)	0.7 (0.5)	0.6 (0.5)	5.9 (4.9)	0.9 (0.7)	6.8 (5.8)
PD post-med	0.3 (0.2)	0.5 (0.4)	0.8 (0.5)	5.4 (4.7)	1.1 (1.4)	6.7 (5.4)
Controls	0.4 (0.2)	0.7 (0.4)	0.7 (0.8)	10.9 (5.3)	1.4 (0.8)	14.1 (5.9)

Gait segment duration was determined based on annotated gait, hence the category non-gait concerns annotated non-gait that did not get detected as such by the classifiers. Groups include the PD group prior to medication intake (pre-med), the PD group after medication intake (post-med), and the control group. The mean (SD) was calculated across individuals. The time shown was for the most affected side only

#### Impact of misclassifications on arm swing parameters

To evaluate the impact of misclassifications in detecting and filtering gait (steps 1 and 2), we assessed the agreement between the arm swing parameters derived from predicted and video-annotated gait segments without other arm activities.

The ICCs are presented in Table 9. Overall, the ICCs for both the median and 95th percentile RoM were high across stratifications, with a moderate ICC observed for the median RoM post-med on the least affected side.

**Table 9:** Intra-class correlation coefficients [95% confidence intervals] for predicted and annotated gait without other arm activities, computed within subjects

Group	Parameter	
	Median RoM	95th percentile RoM
Pre-med MAS	1.00 [0.99, 1.00]	0.99 [0.99, 1.00]
Pre-med LAS	0.97 [0.93, 0.99]	0.99 [0.98, 1.00]
Post-med MAS	0.99 [0.98, 1.00]	0.97 [0.93, 0.99]
Post-med LAS	0.64 [0.26, 0.85]	0.99 [0.97, 1.00]

Results are reported for the median and 95th percentile range of motion (RoM), stratified by medication state (pre-medication (pre-med) versus post-medication (post-med)) and affected side (most affected side (MAS) versus least affected side (LAS))

**Fig. 4:**
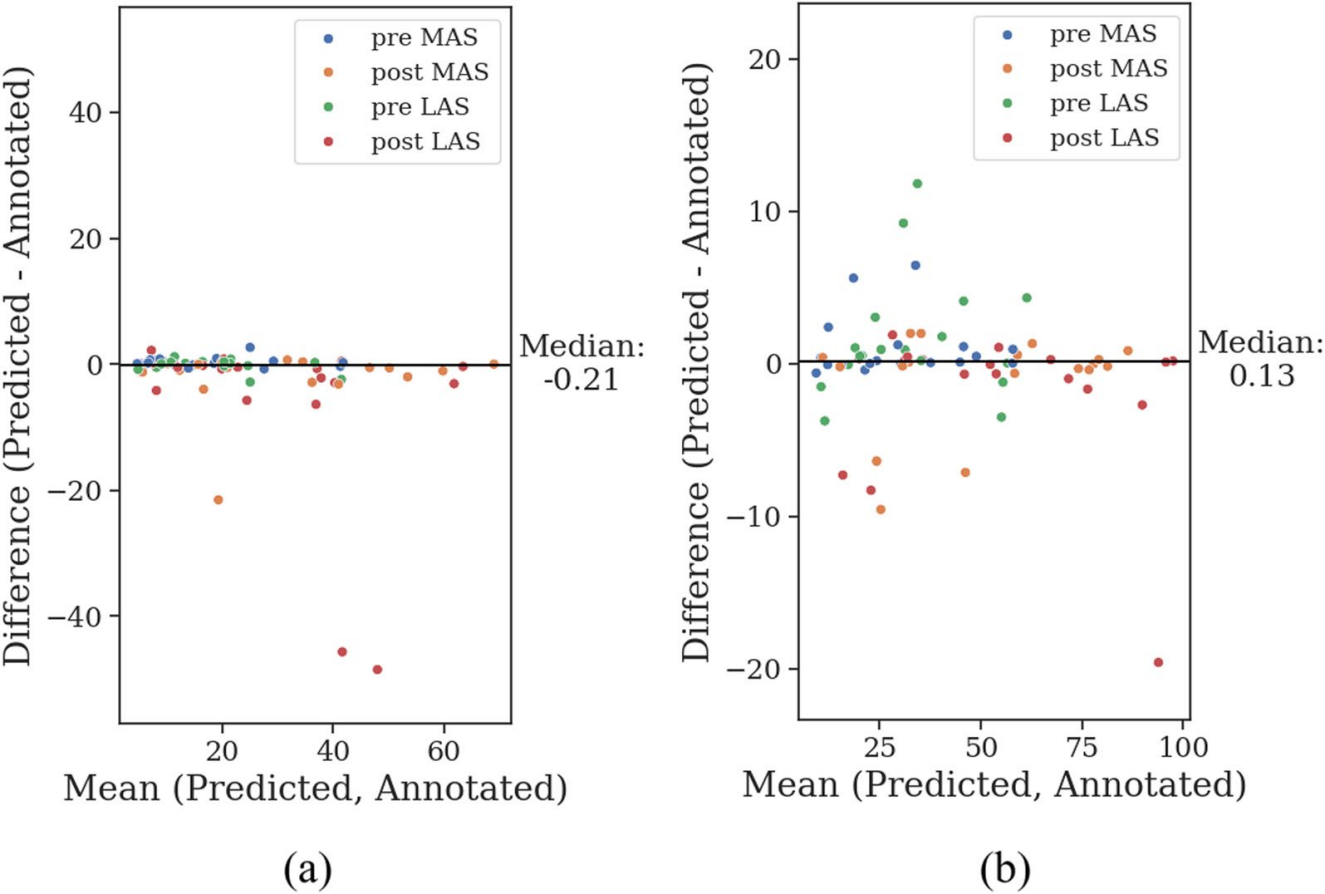
Bland-Altman plots of the **a** median and **b** 95th percentile range of motion. The x-axis represents the mean, and the y-axis represents the difference between the predicted and annotated gait segments without other arm activities. Each data point corresponds to an individual participant with Parkinson’s disease, represented across all combinations of prior to medication intake (pre-med) versus after medication intake (post-med), and most affected side (MAS) versus least affected side (LAS). The horizontal line indicates the median across all participants and combinations

The Bland-Altman plots of predicted versus video-annotated gait without other arm activities are shown in Fig. 4. No evidence of systematic bias was found in either the median or 95th percentile RoM. For both measures, the differences between predicted and annotated gait segments were generally small relative to variability observed between participants in the mean of predicted and annotated segments. Large discrepancies between predicted and annotated segments in post-med were primarily attributed to dyskinesia in the upper limbs, which was classified as other arm activity, leading to an underestimation of the RoM.

#### Sensitivity of arm swing parameters to PD motor signs

We evaluated whether the arm swing parameters of filtered gait segments exhibited expected differences between individuals with PD and controls, between pre-med and post-med, between the most and least affected sides in pre-med, and across varying MDS-UPDRS Part III pre-med subscores.

**Fig. 5:**
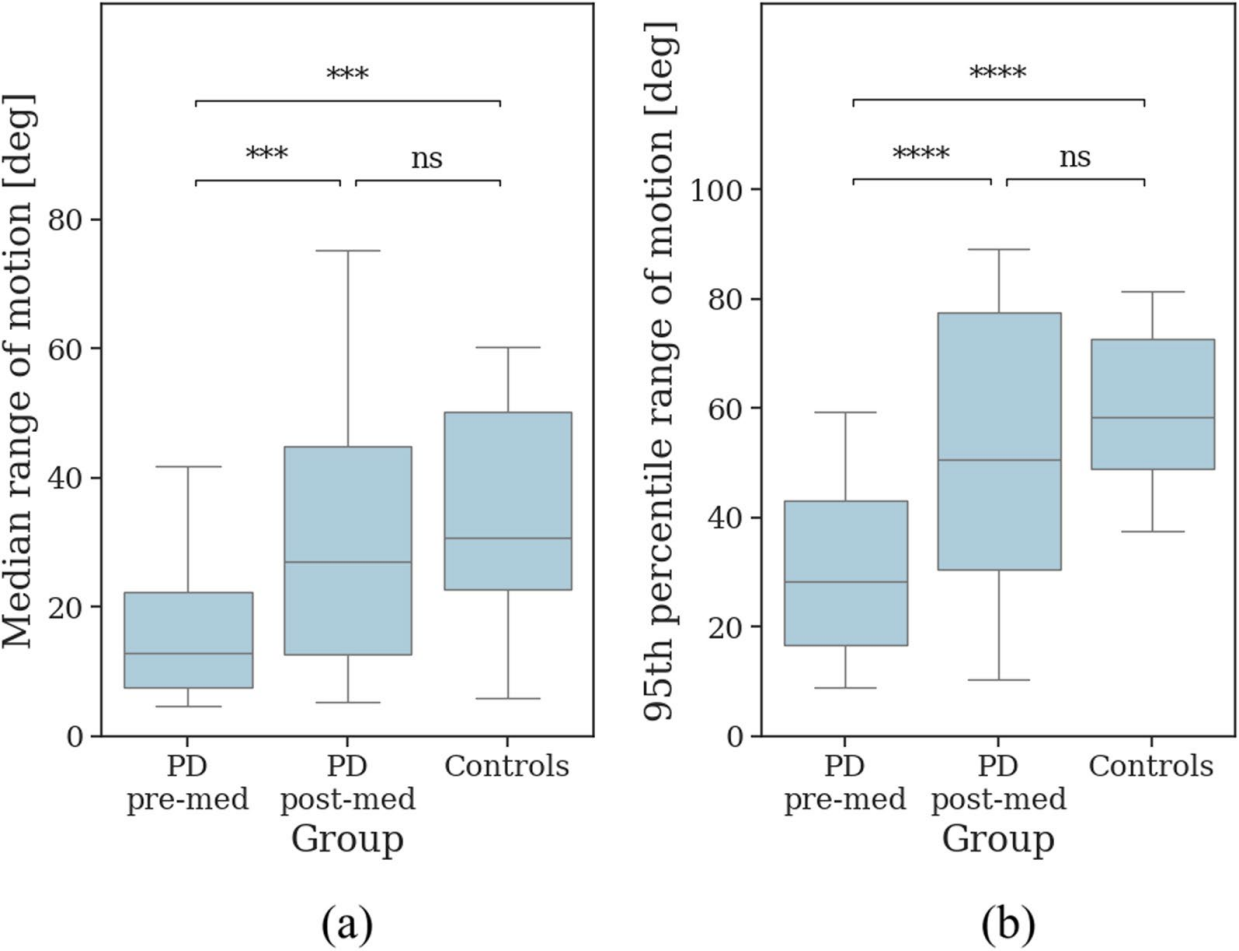
The **a** median and **b** 95th percentile range of motion in filtered gait segments for the most affected side of the PD group prior to medication intake (pre-med), the PD group after medication intake (post-med), and the control group

Fig. 5 shows the median and 95th percentile RoM in filtered gait segments in pre-med, post-med, and controls. Compared to pre-med, controls exhibited a higher median RoM ($\Delta = 18.41^{\circ }$, 95% CI [9.96, 29.86], *p* < 0.001) and 95th percentile RoM ($\Delta = 29.44^{\circ }$, 95% CI [20.18, 40.42], *p* < 0.001). However, compared to post-med, controls did not show a higher median RoM ($\Delta = 7.54^{\circ }$, 95% CI [$-$7.08, 18.03], *p* = 0.26) or 95th percentile RoM ($\Delta = 9.43^{\circ }$, 95% CI [$-$9.08, 26.32], *p* = 0.32).

Both the median RoM ($\Delta = 14.01^{\circ }$, 95% CI [4.80, 22.55], *p* < 0.001) and the 95th percentile RoM ($\Delta = 20.58^{\circ }$, 95% CI [10.81, 29.51], *p* < 0.001) were greater in post-med compared to pre-med. As shown in Figures S14 and S15, the increase in median RoM after medication intake was primarily observed in very long gait segments (short: *p* = 0.08; moderately long: *p* = 0.07; long: *p* = 0.07; very long: *p* < 0.001). For the 95th percentile RoM, increases were noted in short and very long gait segments only (short: *p* = 0.01; moderately long: *p* = 0.13; long: *p* = 0.21; very long: *p* < 0.001).

Figure S16 shows the differences in arm swing parameters between the most and least affected sides. We did not observe a reduction in the median or 95th percentile RoM on the most affected side compared to the least affected side in pre-med (median: $\Delta$ = 0.72$^{\circ }$, 95% CI [$-$8.17, 6.04], *p* = 0.80; 95th percentile: $\Delta$ = 2.31, 95% CI [$-$7.26, 12.00], *p* = 0.65).

**Fig. 6:**
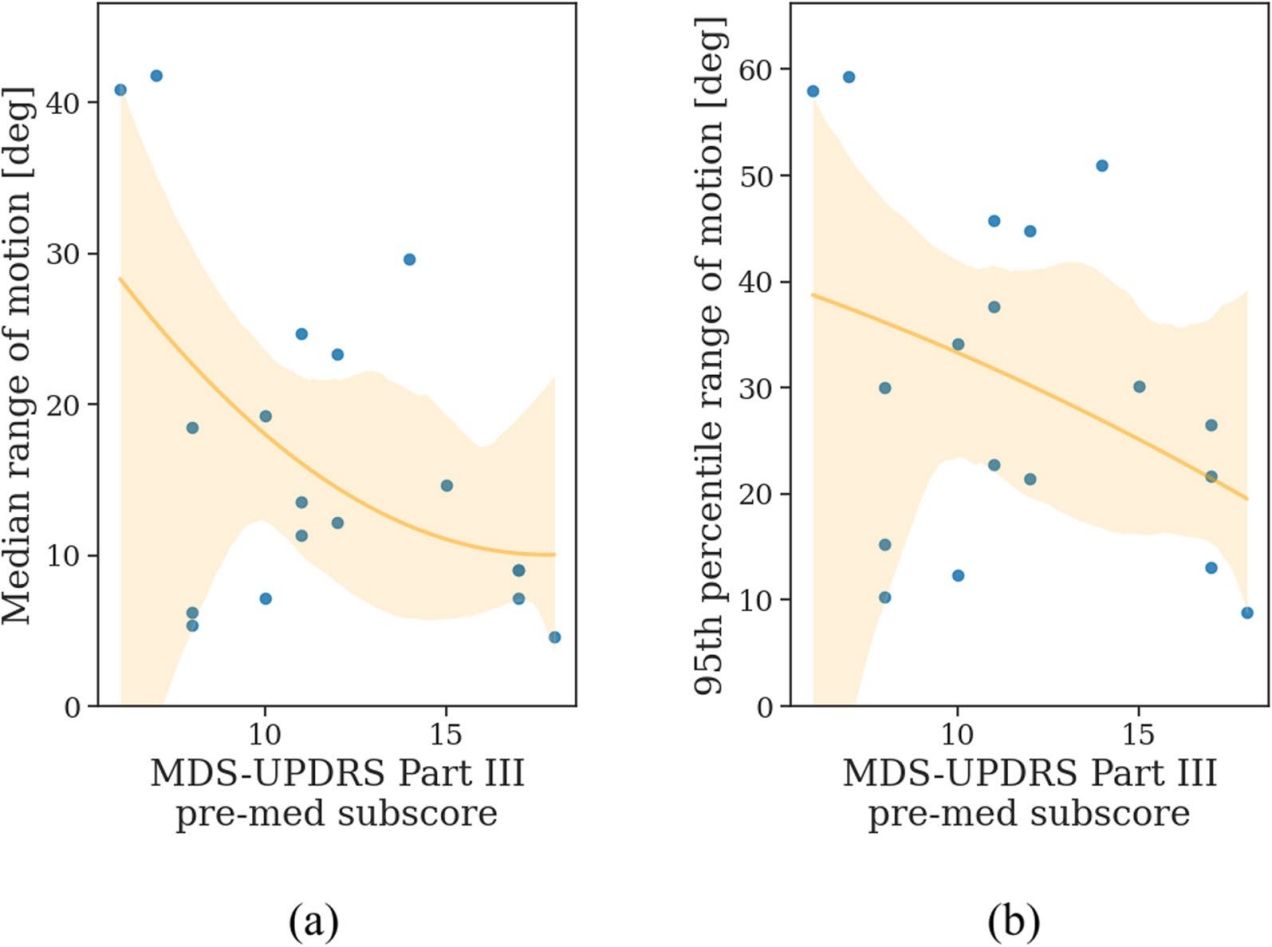
The **a** median and **b** 95th percentile range of motion of filtered gait segments correlated with the MDS-UPDRS Part III pre-medication subscore. Data points represent the aggregated measures for the most affected side of each individual in the PD group prior to medication intake. A second-order polynomial regression fitted to the data points is displayed with a 95% confidence interval in orange

As shown in Fig. 6, individuals with higher MDS-UPDRS Part III pre-med subscores did not exhibit smaller median RoM ($r_{s}$ = $-$0.36, 95% CI [$-$0.71, 0.13], *p* = 0.14) and 95th percentile RoM ($r_{s}$ = $-$0.32, 95% CI [$-$0.68, 0.18], *p* = 0.20).

#### Effect of filtering gait on medication-induced differences in arm swing parameters

To examine the added value of filtering out gait segments with other arm activities, we compared the change in arm swing parameters following dopaminergic medication intake between unfiltered and filtered gait.

**Fig. 7:**
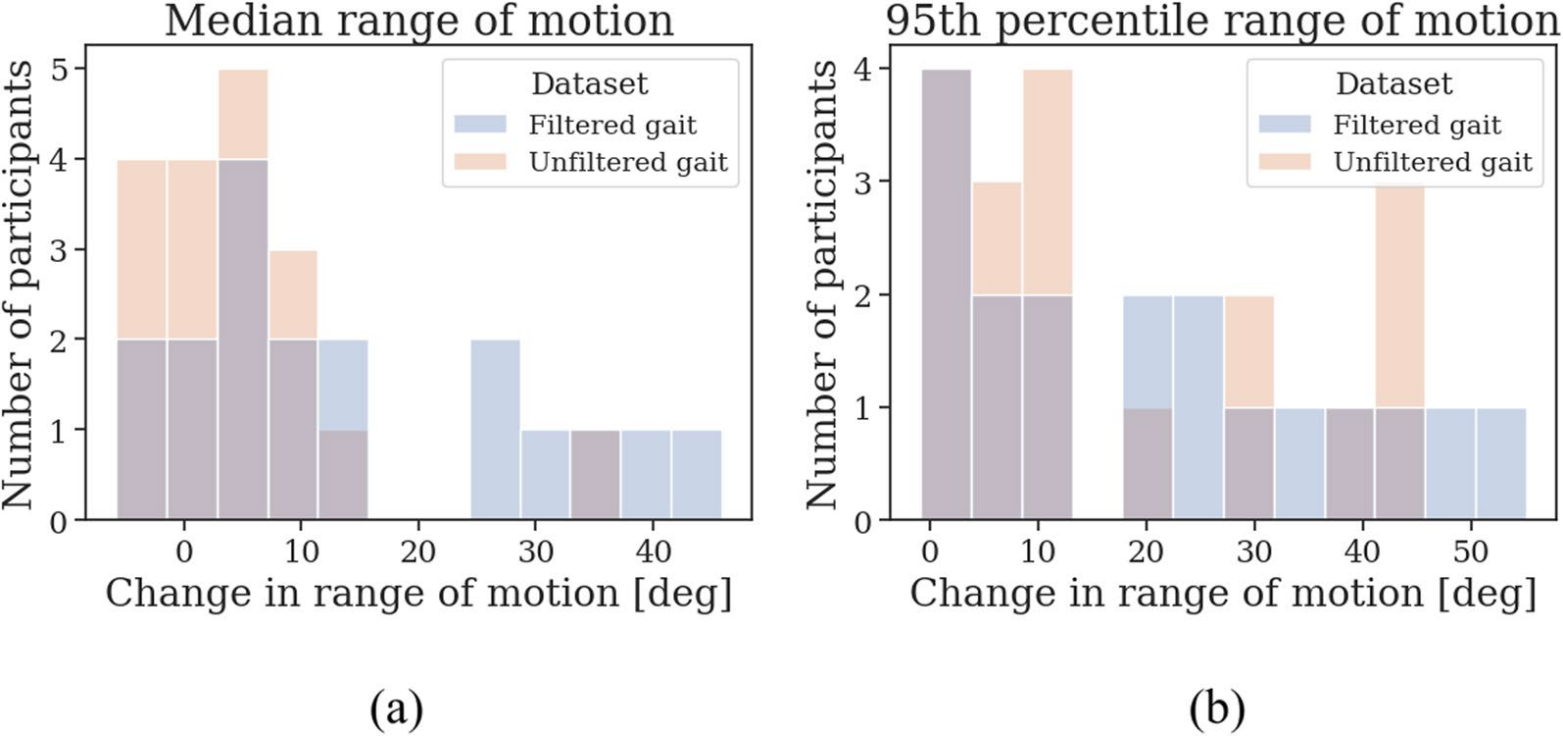
The change in **a** median and **b** 95th percentile range of motion in degrees (deg) after medication intake across PD participants, comparing predicted gait (unfiltered gait) and predicted gait predicted to have no other arm activities (filtered gait)

Fig. 7 displays the distribution of changes in the median and 95th percentile RoM after medication intake for filtered and unfiltered gait. The difference between pre-med and post-med increased after filtering gait for the median RoM ($\Delta$ = 7.92$^{\circ }$, 95% CI [1.25, 14.72], *p* = 0.01) and the 95th percentile RoM ($\Delta$ = 2.13$^{\circ }$, 95% CI [0.04, 5.77], *p* = 0.03).

## Discussion

This study addresses an important challenge in quantifying arm swing during free-living gait using wrist-worn sensors: the variability of other arm activities that occur during gait. By filtering out gait segments with these other arm activities, we aimed to improve the accuracy of arm swing estimations in individuals with PD. Using arm activity annotations from video recordings, we demonstrated a reliable method for filtering gait in this population, independent of medication state. Importantly, our results showed no systematic bias in arm swing RoM estimations by erroneously filtering out arm swings with a smaller RoM, and a high agreement between predicted and annotated gait without other arm activities. As hypothesized, the derived arm swing RoM were reduced in PD compared to controls, and reduced in pre-med compared to post-med. Filtering gait increased the change in both the median and 95th percentile RoM following dopaminergic medication intake, highlighting its potential value for monitoring treatment response in clinical trials.

### Arm swing quantification

Our findings align with in-clinic and active assessment studies that demonstrate the sensitivity of arm swing RoM and other arm swing parameters to the presence of PD and the intake of dopaminergic medication [18–23]. However, the question of whether arm swing increases after dopaminergic medication intake during free-living gait has been debated [5, 22, 24, 33]. For instance, one study reported insensitivity to dopaminergic medication during dual-task gait, which is thought to better simulate real-world conditions [24]. In contrast, we found that dopaminergic medication increased both the median and 95th percentile RoM during unscripted gait at the group level, although responsiveness varied among individuals. This raises questions about the reliability of scripted dual-task gait as a true representation of real-world conditions. Free-living gait may better be characterized as a blend of single- and multi-tasking scenarios influenced by environmental and behavioral factors. Supporting this notion, studies in older adults have indicated that neither single-task nor dual-task gait accurately reflects daily living conditions at the individual level [45]. Additionally, by filtering out gait segments with other arm activities, our analysis focuses more on single-task gait, which may contribute to the observed responsiveness to dopaminergic medication. Further research is necessary to validate these findings in genuinely free-living conditions, as our study still involved assessors accompanying the participants.

Although PD is associated with arm swing asymmetry [46, 47], we found no differences in the arm swing RoM between the most and least affected sides. A possible explanation is that we only included PD patients with response fluctuations, which generally occur later in the disease. In early stages of PD, hypokinesia typically has a strongly asymmetrical presentation [48, 49], but, as the disease progresses, the asymmetry decreases because of increased impairment on the least affected side [50]. Currently, comparing free-living arm swing asymmetry across disease stages is difficult because most studies have focused on task-based gait assessments [21, 24, 29, 47]. Therefore, future work will assess free-living arm swing asymmetry across different disease stages, to examine whether it can be used to measure disease progression.

#### Gait detection

Accurate detection of gait (without other arm activities) is essential for reliably quantifying arm swing during free-living gait. While wrist-worn sensor-based gait detection algorithms are preferred in longitudinal studies due to their low obtrusiveness, they generally underperform compared to those using sensor data from the lower back or ankle [36, 51]. One way to mitigate this performance gap is through advanced machine learning techniques that better capture gait-related subtleties. For instance, self-supervised and deep learning approaches have demonstrated significant improvements in detecting gait using wrist-worn sensor data in individuals with PD [37, 52]. Given the strengths and limitations of our dataset, we opted for a relatively simple supervised classifier. However, the modularity of our pipeline allows for future integration of more advanced methods. Notably, our approach demonstrated no reduced ability to detect gait (without other arm activities) due to PD. Any replacement algorithms must ensure unbiased detection, avoiding the risk of overestimating arm swing in individuals with hypokinetic arm swing.

#### Segment duration

The optimal gait segment duration for assessing the presence of PD and the effect of dopaminergic medication in free-living conditions has long been debated [29, 53, 54]. In our study, we demonstrated a positive correlation between the ability to detect gait (without other arm activities) and gait segment duration. This implies that free-living gait analysis using wrist-worn sensors may naturally focus on the quantification of longer gait segments. This could be beneficial, since we observed differences between pre-med and post-med median and 95th percentile RoM to be most significant in gait segments longer than 20 s. Other studies equivalently showed an increased ability to discriminate between PD and controls when considering longer gait segments [29, 54]. This suggests that longer gait segments may be preferable for both gait detection, filtering gait, and arm swing quantification, although it remains imperative for future work to validate the sensitivity of arm swing parameters in varying gait segment durations in larger cohorts.

#### Strengths

This study marks one of the first explorations of filtering out gait segments with other arm activities. A notable strength of this study is its unscripted nature, particularly important given the scarcity of video-monitored free-living studies. This approach allowed for the annotation of a wide variety of arm activities during gait. However, validation in truly free-living conditions remains essential, as participants’ awareness of being monitored and the presence of assessors could have influenced the participants’ behavior. Another strength of this study is that we made all sensor data and video annotations of activities and arm activities publicly available. This open access allows other researchers to improve or iterate upon the presented methods. The analysis pipeline developed in this study is integrated into the Parkinson’s Disease Digital Markers (ParaDigMa) toolbox [55]. The proposed modular approach to detecting gait, filtering gait and quantifying arm swing enables the comparison of our findings with other gait detection research, and facilitates the substitution of the implemented methods with more advanced techniques in future studies.

#### Limitations

This study carries several limitations that warrant discussion. First, the sample of 25 PD participants was relatively small, and larger studies are needed to better capture the heterogeneity of PD. For example, we observed considerable variability in the model’s ability to correctly classify gait without other arm activities, both across types of arm activities and among individual participants. Similarly, the small sample size limits the reliability of the increased difference between pre-med and post-med range of motion after filtering gait. In response, to validate the proposed methodology, it is necessary to assess the classifiers’ variability in larger-scale studies across diverse cohorts and settings, and to verify the reliability across subsequent weeks. Both the validity and reliability we aim to address in upcoming work. Importantly, despite the model’s reduced accuracy in detecting gait without other arm activities in participants with relatively high MDS-UPDRS Part III subscores, we found no systematic overestimation of arm swing parameters. Secondly, our model’s sensitivity to dyskinesias presents another limitation. Our dataset included only one participant with mild dyskinesias (25–50% of the waking day) and five with slight dyskinesias (0–25% of the waking day), likely due to the adverse effects of dopaminergic medication. In the individual with mild dyskinesias, these episodes were often misclassified as gait with other arm activities, leading to notable discrepancies in the RoM between video-annotated and predicted gait segments without other arm activities. More data from individuals with prevalent dyskinesias are essential for accurately distinguishing these from voluntary arm activities during gait. Furthermore, further research is needed to determine whether complex and fluctuating motor patterns, such as dyskinesias, can be reliably captured using a single wrist-worn sensor [56].

Despite these limitations, the findings of this study provide important insights into the potential for using the median and 95th percentile RoM as indicators of dopaminergic treatment response. Our upcoming work will continue the validation by assessing the suitability of these measures for longitudinally tracking disease progression. This is an imperative next step toward the acceptance of these arm swing measures as digital biomarkers in clinical trials investigating disease-modifying therapies.

## Conclusion

Using a video-referenced dataset of unscripted daily activities, we developed a method to isolate gait segments free from concurrent arm activities. This enables a more specific estimation of arm swing range of motion in free-living conditions using wrist-worn sensors. We show that this approach can be used for monitoring PD-related changes in arm swing, offering a tool to monitor the course of PD and response to dopaminergic treatment in daily life.

## Supplementary Information

**Supplementary Material 1.**

## Data Availability

The data and other supporting materials of this study are publicly available in the Radboud Data Repository: 10.34973/fr4z-a489. To concur with privacy standards, video recordings of the participants were excluded from the repository. The code used to generate the results in this study is publicly available in the Git repository: https://github.com/biomarkersParkinson/pdathome_gait. The repository is built upon the ParaDigMa toolbox: 10.5281/zenodo.13838392. Installing all library dependencies can conveniently be done using Poetry, the environment manager used in this repository.

## Abbreviations

CI: Confidence interval
HC: Healthy controls
ICC: Intra-class correlation coefficient
LAS: Least affected side
LASSO: Least absolute shrinkage selection operator
LOSO-CV: Leave-one-subject-out cross-validation
LR: Logistic regression
MAS: Most affected side
MDS-UPDRS: Movement Disorders Society-sponsored Unified Parkinson’s Disease Rating Scale
PD: Parkinson’s disease
RBD: Rapid eye movement sleep behavior disorder
RF: Random forest
RoM: Range of motion
SD: Standard deviation
