# Supplementary material for: Quantifying arm swing in Parkinson’s disease: a method accounting for arm activities during free-living gait
Source: J Neuroeng Rehabil. 2025 Feb 26;22:37. doi: 10.1186/s12984-025-01578-z (PMC11863854; doi:10.1186/s12984-025-01578-z)
Supplement: Supplementary file 1 — Supplementary Material 1. [file 12984_2025_1578_MOESM1_ESM.pdf]

# Supplementary

**Table S1**: Characteristics of individuals constituting the PD cohort.

| Characteristics | | | MDS-UPDRS | | AIMS | | H&Y stage | |
|---|---|---|---|---|---|---|---|---|
| ID | Age | YSD† | 3-10 Off | 3-10 On | 5 Off | 5 On | Off | On |
| 002 | 71 | 22 | 2 | 1 | N/A | 0 | 2 | 2 |
| 012 | 63 | 13 | 1 | 1 | N/A | 0 | 1 | 1 |
| 013 | 74 | 15 | 2 | 1 | N/A | 0 | 2 | 2 |
| 014 | 73 | 10 | 1 | 1 | N/A | 0 | 2 | 2 |
| 015 | 76 | 16 | 2 | 1 | N/A | 3 | 3‡ | 2 |
| 016 | 77 | 10 | 1 | 1 | N/A | 0 | 2 | 2 |
| 017 | 64 | 11 | 2 | 1 | N/A | 0 | 2 | 2 |
| 018 | 74 | 13 | 0 | 0 | N/A | 0 | 2 | 2 |
| 022 | 68 | 11 | 1 | 1 | N/A | 0 | 2 | 2 |
| 023 | 64 | 9 | 2 | 1 | 2 | 0 | 4 | 3 |
| 024 | 57 | 10 | 2 | 2 | N/A | 0 | 2 | 2 |
| 038 | 54 | 10 | 2 | 1 | N/A | 0 | 2 | 2 |
| 039 | 74 | 15 | 1 | 1 | N/A | 0 | 2 | 2 |
| 043 | 66 | 15 | 1 | 0 | N/A | 0 | 2 | 1 |
| 047 | 73 | 9 | 2 | 1 | N/A | 0 | 3 | 2 |
| 054 | 66 | 10 | 0 | 0 | N/A | 0 | 3 | 3 |
| 058 | 77 | 17 | 2 | 2 | 1 | 1 | 2 | 3 |
| 063 | 66 | 9 | 1 | 0 | N/A | 0 | 2 | 2 |
| 065 | 66 | 16 | 2 | 2 | N/A | 0 | 2 | 2 |
| 077 | 61 | 12 | 1 | 0 | N/A | 0 | 2 | 2 |
| 079 | 71 | 12 | 1 | 1 | N/A | 0 | 3 | 3 |
| 090 | 77 | 18 | 1 | 1 | N/A | 0 | 2 | 2 |

† YSD = Years since diagnosis.
‡ The H&Y state pre-medication was determined based on video recordings for this participant since the physical assessment was missing.

Scores include the Movement Disorder Society Unified Parkinson Disease Rating Scale (MDS-UPDRS) item 3.10 for gait impairments, the Abnormal Involuntary Movement Rating Scale (AIMS) item 5 for dyskinesia, and the Hoehn & Yahr scale (H&Y) for disease state. The scores are provided for the pre-medication (off) and post-medication (on) states. The AIMS scores were not assessed for controls and are hence denoted by not applicable (N/A).

**Table S2**: Distribution of the number of participants performing specific activities.

| | PD | | Controls | |
|---|---|---|---|---|
| Activity | n (%) | Mean (SD) | n (%) | Mean (SD) |
| All | 23 (100) | 154.1 (28.5) | 24 (100) | 95.3 (17.6) |
| Sitting | 23 (100) | 91.8 (26.7) | 24 (100) | 48.7 (14.1) |
| Standing | 23 (100) | 26.5 (14.4) | 24 (100) | 22.3 (9.1) |
| Walking | 23 (100) | 25.4 (10.5) | 24 (100) | 19.8 (6.5) |
| Lying | 5 (22) | 0.0 (0.1) | 4 (17) | 0.0 (0.1) |
| Unknown | 23 (100) | 3.5 (2.5) | 24 (100) | 1.1 (1.0) |
| Turning | 23 (100) | 1.0 (0.5) | 24 (100) | 0.6 (0.4) |
| Transition | 23 (100) | 0.9 (0.4) | 24 (100) | 0.5 (0.2) |
| Walking the stairs | 19 (83) | 0.7 (0.5) | 24 (100) | 0.6 (0.4) |
| Cycling | 4 (17) | 0.5 (1.3) | 12 (50) | 0.6 (0.7) |
| Crosstrainer | 3 (13) | 0.2 (0.4) | 0 (0) | 0.0 (0.0) |
| Push-ups | 0 (0) | 0.0 (0.0) | 1 (4) | 0.0 (0.1) |
| Driving a vehicle | 0 (0) | 0.0 (0.0) | 2 (8) | 0.9 (4.0) |
| Running | 0 (0) | 0.0 (0.0) | 1 (4) | 0.0 (0.1) |

For each activity, the number (n) and percentage (%) of participants performing the activity, along with the mean (SD) duration of the activity in minutes across participants.

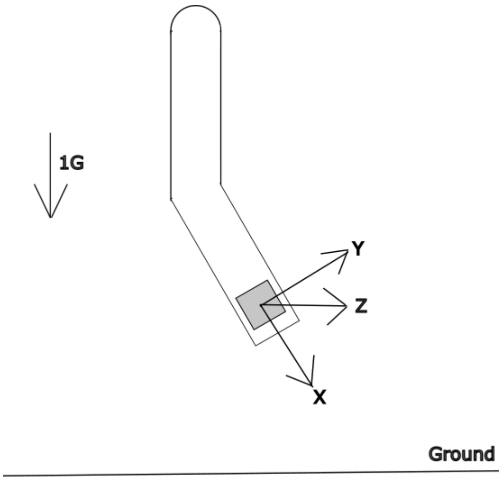


**Fig. S1**: Accelerometer axes are transformed in accordance to the axes directions shown in this figure. If the vector of an axis is pointing downward along to the force of gravity (downward arrow of 1G) and the sensor is stable (i.e., there is no acceleration due to movement), the corresponding acceleration equals -1G in this axis. The gyroscope axes are transformed in accordance with the accelerometer axes by applying the right-hand screw rule.

**Table S3**: Distribution of the number of participants performing specific arm activities.

| | Most affected side | | Least affected side | |
|---|---|---|---|---|
| Arm activity | n (%) | Mean (SD) | n (%) | Mean (SD) |
| All | 23 (100) | 25.4 (10.6) | 23 (100) | 25.4 (10.6) |
| Arm swing | 23 (100) | 12.6 (8.5) | 23 (100) | 12.7 (9.8) |
| Holding dog leash | 6 (26) | 4.7 (4.1) | 5 (22) | 5.9 (7.8) |
| Hand in front trouser pocket | 8 (35) | 5.6 (6.8) | 6 (26) | 3.0 (7.4) |
| Vacuum cleaning | 1 (4) | 3.4 (0.0) | 1 (4) | 2.9 (0.0) |
| Mowing lawn | 1 (4) | 2.7 (0.0) | 1 (4) | 2.7 (0.0) |
| Holding object forward | 23 (100) | 2.4 (1.2) | 23 (100) | 2.2 (1.1) |
| Hand in jacket pocket | 7 (30) | 1.8 (2.0) | 10 (43) | 2.8 (2.5) |
| Holding object downward | 14 (61) | 1.2 (4.0) | 11 (48) | 0.2 (0.3) |
| Unknown | 23 (100) | 1.1 (0.7) | 23 (100) | 1.1 (0.7) |
| Hand on waist | 11 (48) | 0.8 (1.8) | 10 (43) | 0.8 (2.0) |
| Making hand gestures | 20 (87) | 0.6 (0.9) | 22 (96) | 0.7 (1.1) |
| Hands forward | 12 (52) | 0.6 (0.8) | 14 (61) | 0.5 (0.5) |
| Hand on trouser pocket | 8 (35) | 0.5 (0.8) | 10 (43) | 0.3 (0.7) |
| Grabbing | 23 (100) | 0.5 (0.4) | 23 (100) | 0.6 (0.4) |
| Pushing forward | 5 (22) | 0.4 (0.9) | 7 (30) | 0.4 (0.9) |
| Pulling backward | 6 (26) | 0.3 (0.6) | 7 (30) | 0.3 (0.6) |
| Holding forward and rotating | 15 (65) | 0.3 (0.3) | 15 (65) | 0.3 (0.2) |
| Patting pet | 3 (13) | 0.3 (0.4) | 3 (13) | 0.2 (0.2) |
| Hand clasped in front | 1 (4) | 0.3 (0.0) | 1 (4) | 0.3 (0.0) |
| Using hand for support | 22 (96) | 0.3 (0.2) | 23 (100) | 0.4 (0.3) |
| Putting on dog leash | 2 (9) | 0.3 (0.1) | 2 (9) | 0.3 (0.2) |
| Brushing teeth | 1 (4) | 0.3 (0.0) | 0 (0) | 0.0 (0.0) |
| Fixing clothes | 16 (70) | 0.2 (0.2) | 16 (70) | 0.2 (0.3) |
| Hands folded across | 2 (9) | 0.2 (0.2) | 1 (4) | 0.0 (0.0) |
| Opening by pulling | 19 (83) | 0.2 (0.2) | 23 (100) | 0.3 (0.2) |
| Walking stairs | 1 (4) | 0.2 (0.0) | 1 (4) | 0.2 (0.0) |
| Touching face | 14 (61) | 0.2 (0.2) | 21 (91) | 0.2 (0.2) |
| Pointing | 21 (91) | 0.1 (0.2) | 19 (83) | 0.3 (0.6) |
| Putting on jacket | 9 (39) | 0.1 (0.1) | 9 (39) | 0.1 (0.1) |
| Closing by pulling | 18 (78) | 0.1 (0.1) | 14 (61) | 0.1 (0.2) |
| Taking off jacket | 9 (39) | 0.1 (0.1) | 9 (39) | 0.1 (0.1) |
| Opening by pushing | 15 (65) | 0.1 (0.1) | 17 (74) | 0.2 (0.1) |
| Hand in back trouser pocket | 5 (22) | 0.1 (0.1) | 5 (22) | 0.0 (0.0) |
| Hand on chest | 7 (30) | 0.1 (0.1) | 6 (26) | 0.1 (0.1) |
| Turning on/off lights | 11 (48) | 0.1 (0.1) | 11 (48) | 0.1 (0.1) |
| Grabbing from pocket | 6 (26) | 0.1 (0.1) | 7 (30) | 0.1 (0.1) |
| Transitioning | 20 (87) | 0.0 (0.0) | 20 (87) | 0.0 (0.1) |
| Scratching back | 1 (4) | 0.0 (0.0) | 1 (4) | 0.0 (0.0) |
| Cleaning with cloth | 1 (4) | 0.0 (0.0) | 1 (4) | 0.0 (0.0) |
| Closing by pushing | 13 (57) | 0.0 (0.0) | 11 (48) | 0.0 (0.0) |
| Running | 1 (4) | 0.0 (0.0) | 1 (4) | 0.0 (0.0) |
| Calling with phone | 0 (0) | 0.0 (0.0) | 2 (9) | 0.2 (0.1) |
| Pulling behind back | 0 (0) | 0.0 (0.0) | 3 (13) | 0.5 (0.4) |

For each arm activity, the number (n) and percentage (%) of PD participants performing the arm activity, along with the mean (SD) duration of the arm activity in minutes for each side (most affected and least affected) across PD participants.

**Table S4**: The list of MDS-UPDRS Part III categories included in the composed MDS-UPDRS Part III subscore.

| Item | Name |
|---|---|
| Right Side | |
| 3.3b | Rigidity right upper extremity |
| 3.4a | Finger tapping right hand |
| 3.5a | Movement right hand |
| 3.6a | Pronation-supination right hand |
| 3.7a | Toe tapping right foot |
| 3.8a | Leg agility right leg |
| Left Side | |
| 3.3c | Rigidity left upper extremity |
| 3.3d | Rigidity right lower extremity |
| 3.4b | Finger tapping left hand |
| 3.5b | Movement left hand |
| 3.6b | Pronation-supination left hand |
| 3.7b | Toe tapping left foot |
| 3.8b | Leg agility left leg |

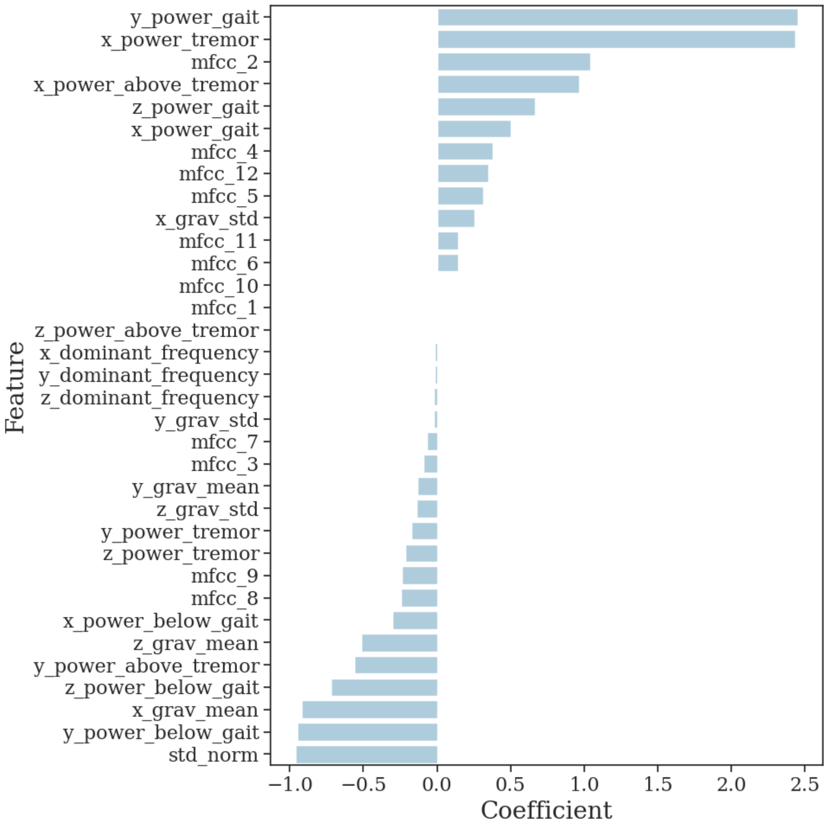


**Fig. S2**: Feature coefficients of the logistic regression classifier in the gait detection step. The features include aggregates of the gravitational component of acceleration (*grav*), Mel Frequency Cepstral Coefficients (*mfcc*), the dominant frequency, the standard deviation of the norm (*std norm*), and the power in specific frequency bands: below gait band ($<$ 0.7 Hz), gait band (0.7–3.5 Hz), tremor band (3.5–8 Hz), and above tremor band ($\geq$ 8 Hz). All features were extracted from the accelerometer signal.

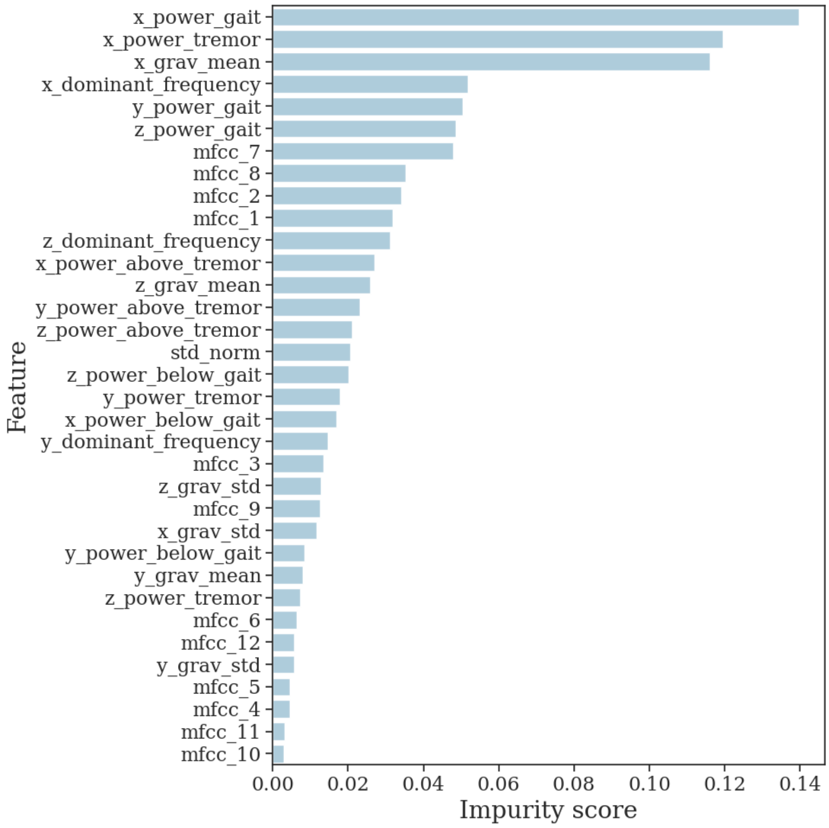


**Fig. S3**: Feature impurity score of the random forest classifier in the gait detection step. The features include aggregates of the gravitational component of acceleration (*grav*), Mel Frequency Cepstral Coefficients (*mfcc*), the dominant frequency, the standard deviation of the norm (*std norm*), and the power in specific frequency bands: below gait band ($< 0.7$ Hz), gait band (0.7–3.5 Hz), tremor band (3.5–8 Hz), and above tremor band ($\geq 8$ Hz). All features were extracted from the accelerometer signal.

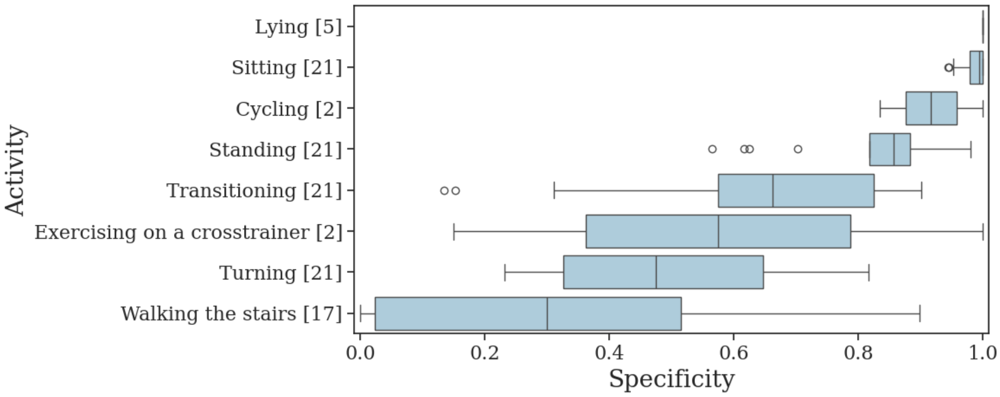


**Fig. S4**: Gait detection specificity at mean 0.89 (SD 0.09) sensitivity, stratified by activities for the most affected side of PD participants prior to medication intake. The number in square brackets indicates the total number of participants who engaged in each activity.

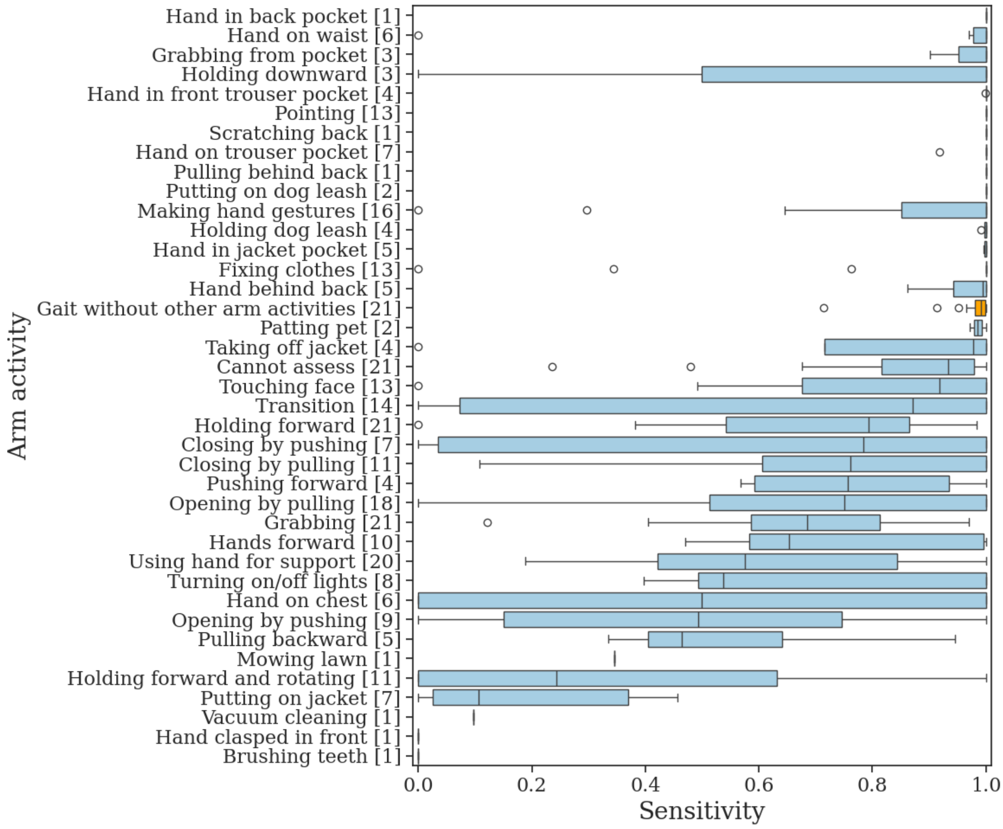


**Fig. S5**: Gait detection sensitivity at mean 0.95 (SD 0.03) specificity, stratified by arm activities for the most affected side of PD participants prior to medication intake. The number in square brackets indicates the total number of participants engaging in each activity. Gait without other arm activities is highlighted in orange.

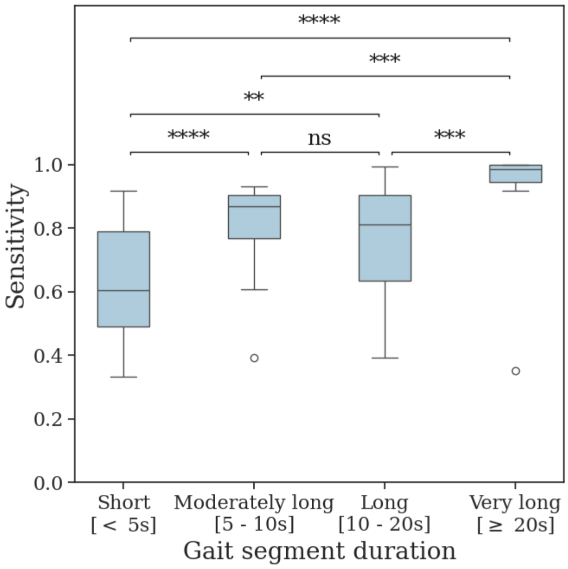


**Fig. S6**: Gait detection sensitivity at 0.95 (0.03) specificity, stratified by segment duration for the most affected side of PD participants prior to medication intake. The outlier in gait segments of very long duration ($\geq 20$ s) is due the large proportion of gait data containing arm activities related to vacuum cleaning, which were annotated as gait.

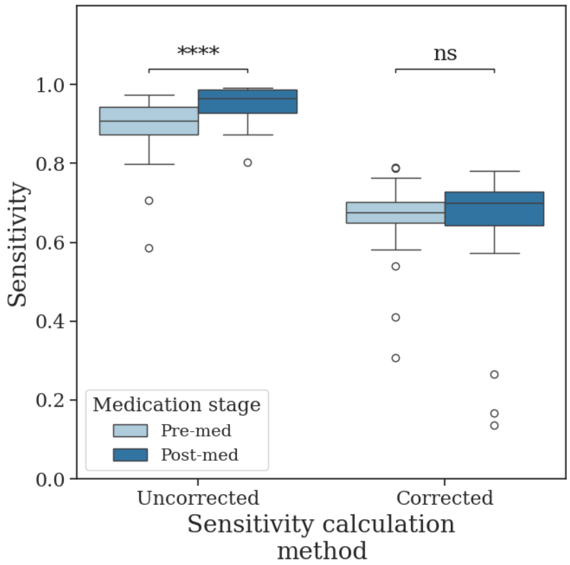


**Fig. S7**: Comparison between the sensitivity of the gait detection classifier for the most affected side of PD participants prior to (pre-med) and after medication intake (post-med). Lower pre-med sensitivity may be influenced by factors such as variation in distributions or hypokinesia, with the latter potentially introducing model bias. Sensitivity differences between pre-med and post-med are presented with and without corrections for arm activity and segment duration. After adjusting for these factors, the classifier's sensitivity was not higher post-med than pre-med ($\Delta = 0.00°$, 95% CI [-0.04, 0.07], $p = 0.87$), suggesting that the observed differences were primarily due to variation in distributions.

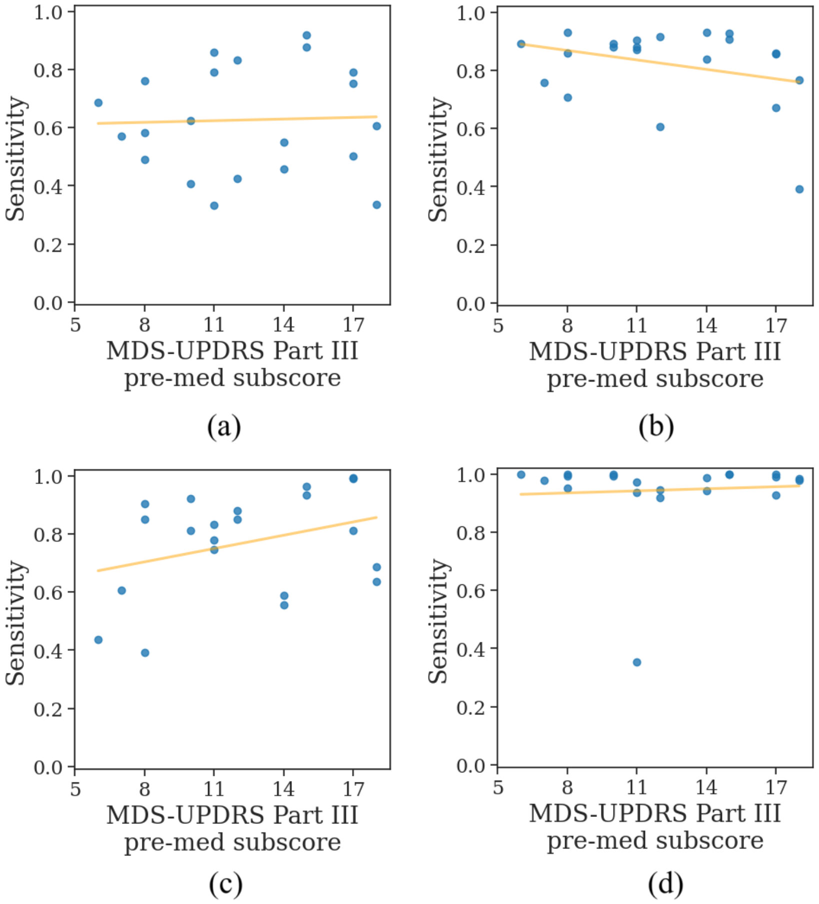


**Fig. S8**: Spearman correlation ($r_s$) between the MDS-UPDRS Part III pre-medication (pre-med) subscore and the sensitivity of the gait detection classifier for the most affected side of PD participants, pre-medication. The correlations are stratified by gait segment duration, with (a) short segments ($< 5$ s), (b) moderately long segments (5–10 s), (c) long segments (10–20 s), and very long segments ($\geq 20$ s). The classifier did not have a lower sensitivity for participants with a higher MDS-UPDRS Part III pre-med subscore across any segment duration: short ($r_s$ = 0.07, $p$ = 0.78), moderately long ($r_s$ = -0.23, $p$ = 0.31), long ($r_s$ = 0.29, $p$ = 0.20), and very long ($r_s$ = -0.09, $p$ = 0.71).

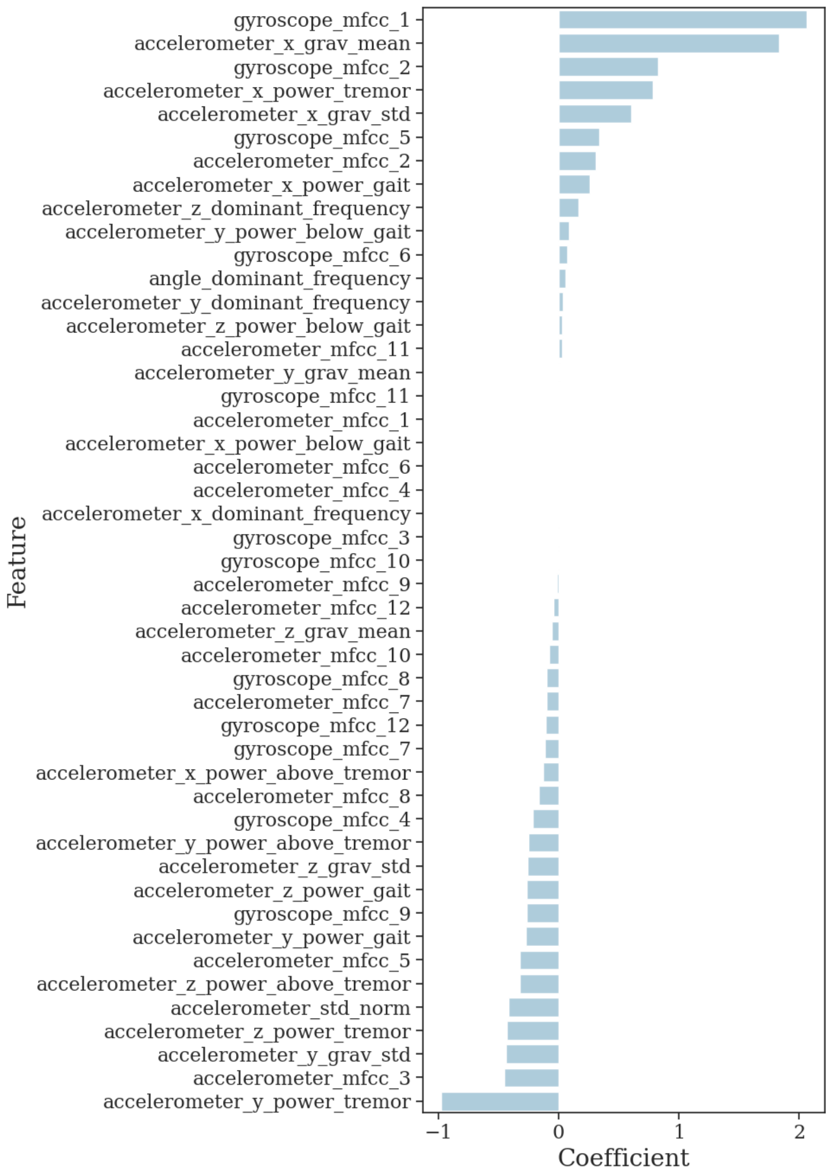


**Fig. S9**: Feature coefficients of the logistic regression classifier detecting gait without other arm activities during gait. Features include the power in specific frequency bands (*power*: *below gait* band [< 0.7 Hz], *gait* band [0.7–3.5 Hz], *tremor* band [3.5–8 Hz], and *above tremor* band [≥ 8 Hz]), gravitation (*grav*), Mel Frequency Cepstral Coefficients (*mfcc*), dominant frequency, and standard deviation of the norm (*std norm*). Features were extracted from the accelerometer and gyroscope signal.

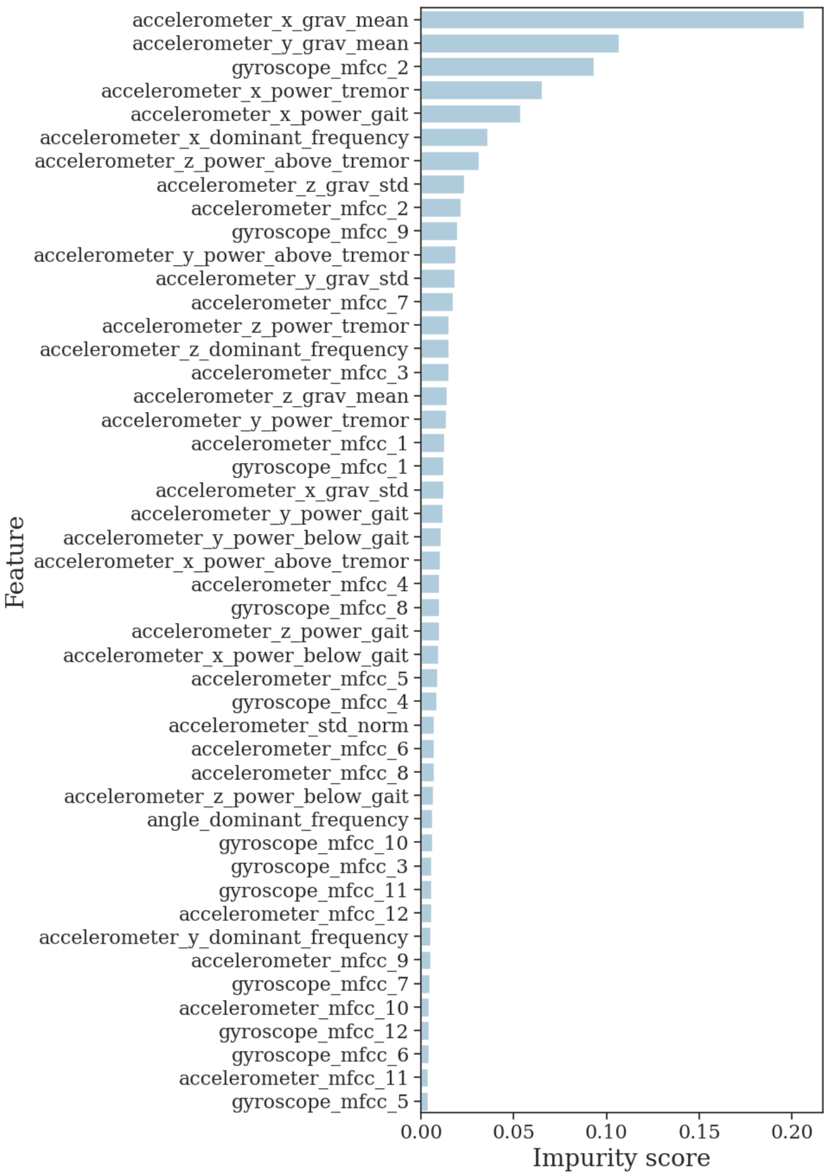


**Fig. S10**: Feature impurity score of the random forest classifier detecting gait without other arm activities during gait. Features include the power in specific frequency bands (*power*: *below gait* band [< 0.7 Hz], *gait* band [0.7–3.5 Hz], *tremor* band [3.5–8 Hz], and *above tremor* band [≥ 8 Hz]), gravitation (*grav*), Mel Frequency Cepstral Coefficients (*mfcc*), dominant frequency, and standard deviation of the norm (*std norm*). Features were extracted from the accelerometer and gyroscope signal.

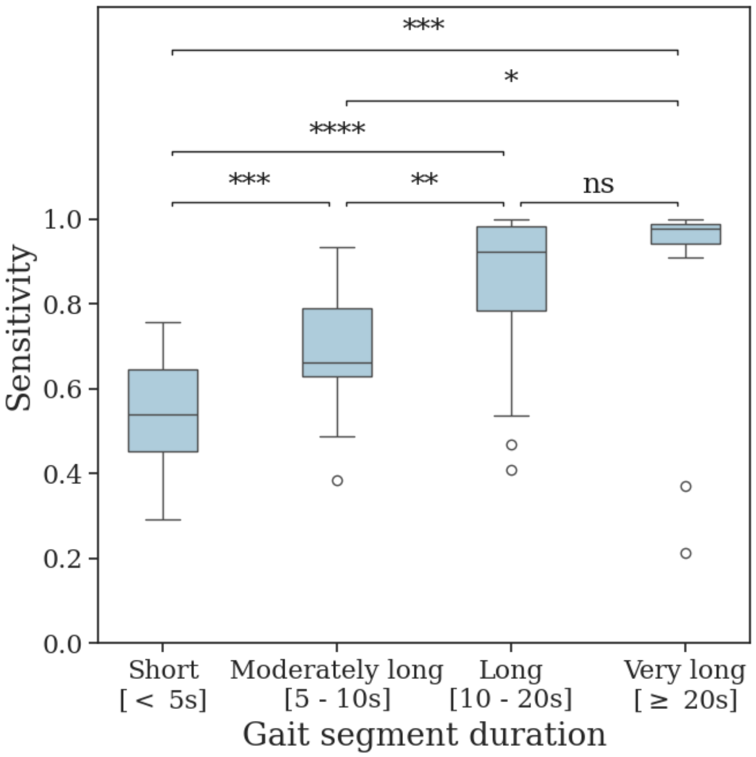


**Fig. S11**: Sensitivity of the classifier detecting gait without other arm activities in varying gait segment durations.

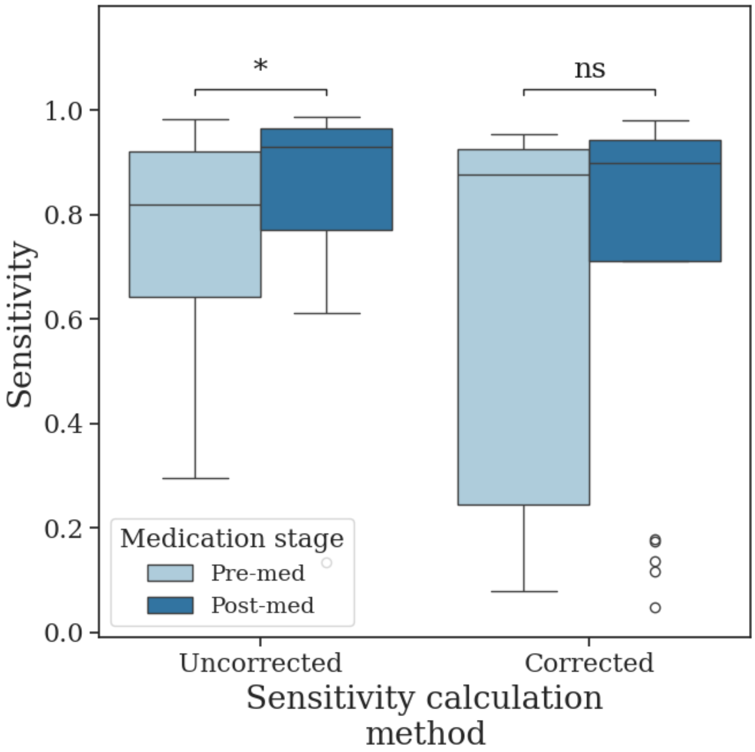


**Fig. S12**: Comparison between the sensitivity of detecting gait without other arm activities for the most affected side of PD participants prior to medication intake (pre-med) compared with after medication intake (post-med). Lower sensitivity may be influenced by factors such as variation in distributions or hypokinesia, with the latter potentially introducing model bias. Sensitivity differences between pre-med and post-med are shown with and without correction for segment duration. After correcting, the sensitivity of the classifier was not higher for post-med than for pre-med ($\Delta = 0.02°$, 95% CI [-0.03, 0.06], $p = 0.29$), indicating that the observed differences were primarily due to variation in distributions.

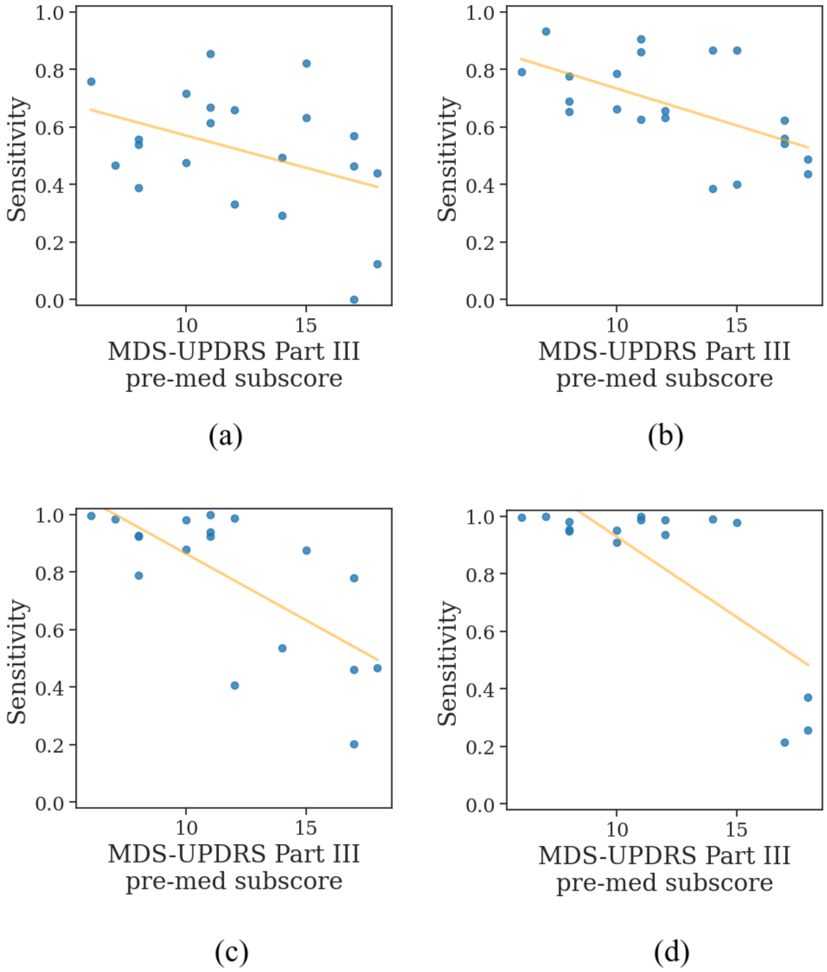


**Fig. S13**: Correlation ($r_s$) between the MDS-UPDRS Part III pre-medication (pre-med) subscore and the sensitivity of detecting gait without other arm activities for the most affected side of PD participants pre-med. The correlations are stratified by gait segment duration, with (a) short segments (< 5 s), (b) moderately long segments (5–10 s), (c) long segments (10–20 s), and very long segments ($\geq$ 20 s). Individuals with a higher MDS-UPDRS Part III pre-med subscore showed a lower sensitivity in moderately long segments ($r_s$ = -0.62, 95% CI [-0.83, -0.26], $p < 0.01$), long segments ($r_s$ = -0.67, 95% CI [-0.87, -0.30], $p < 0.01$), and in very long segments ($r_s$ = -0.51, 95% CI [-0.81, -0.03], $p = 0.04$). We did not observe this correlation in short segments ($r_s$ = -0.34, 95% CI [-0.67, 0.11], $p = 0.13$).

**Table S5**: The sensitivity (sens) and specificity (spec) of the selected classifiers of gait detection and filtering gait for each PD participant (ID) prior to medication intake (pre-med) and after medication intake (post-med).

| | Gait detection | | | | Filtering gait | | | |
|---|---|---|---|---|---|---|---|---|
| | Pre-med | | Post-med | | Pre-med | | Post-med | |
| ID | Sens | Spec | Sens | Spec | Sens | Spec | Sens | Spec |
| 002 | 0.89 | 0.96 | 0.95 | 0.95 | 0.92 | 0.90 | 0.97 | 0.93 |
| 012 | 0.85 | 0.90 | 0.88 | 0.90 | 0.82 | 0.95 | 0.77 | 0.93 |
| 013 | 0.71 | 0.97 | 0.87 | 0.94 | 0.44 | 0.91 | 0.94 | 0.89 |
| 014 | 0.91 | 0.95 | 0.92 | 0.96 | 0.90 | 0.82 | 0.93 | 0.94 |
| 015 | 0.87 | 0.93 | 0.99 | 0.89 | 0.46 | 0.91 | 0.72 | 0.95 |
| 016 | 0.91 | 0.96 | 0.93 | 0.95 | 0.74 | 0.93 | 0.86 | 0.82 |
| 017 | 0.86 | 0.93 | 0.97 | 0.96 | 0.30 | 0.98 | 0.13 | 0.99 |
| 018 | 0.97 | 0.88 | 0.99 | 0.91 | 0.64 | 0.89 | 0.69 | 0.94 |
| 022 | 0.94 | 0.97 | 0.97 | 0.86 | 0.88 | 0.94 | 0.94 | 0.94 |
| 023 | 0.96 | 0.92 | 0.99 | 0.91 | 0.91 | 0.90 | 0.92 | 0.82 |
| 024 | 0.94 | 0.96 | 0.97 | 0.96 | 0.31 | 0.93 | 0.93 | 0.87 |
| 038 | 0.59 | 0.95 | 0.94 | 0.89 | 0.89 | 0.94 | 0.96 | 0.60 |
| 039 | 0.95 | 0.97 | 0.99 | 0.96 | 0.51 | 0.99 | 0.94 | 0.94 |
| 043 | 0.87 | 0.97 | 0.96 | 0.95 | 0.81 | 0.88 | 0.82 | 0.91 |
| 054 | 0.80 | 0.97 | 0.80 | 0.95 | 0.73 | 0.97 | 0.61 | 0.96 |
| 058 | 0.90 | 0.98 | 0.98 | 0.96 | 0.93 | 0.98 | 0.99 | 0.95 |
| 063 | 0.90 | 0.93 | 0.95 | 0.87 | 0.95 | 0.95 | 0.97 | 0.89 |
| 065 | 0.92 | 0.97 | 0.90 | 0.97 | 0.82 | 0.93 | 0.76 | 0.90 |
| 077 | 0.97 | 0.96 | 0.99 | 0.77 | 0.98 | 0.84 | 0.97 | 0.93 |
| 079 | 0.94 | 0.91 | 0.97 | 0.86 | 0.94 | 0.86 | 0.97 | 0.89 |
| 090 | 0.92 | 0.97 | 0.99 | 0.84 | 0.98 | 0.89 | 0.98 | 0.86 |

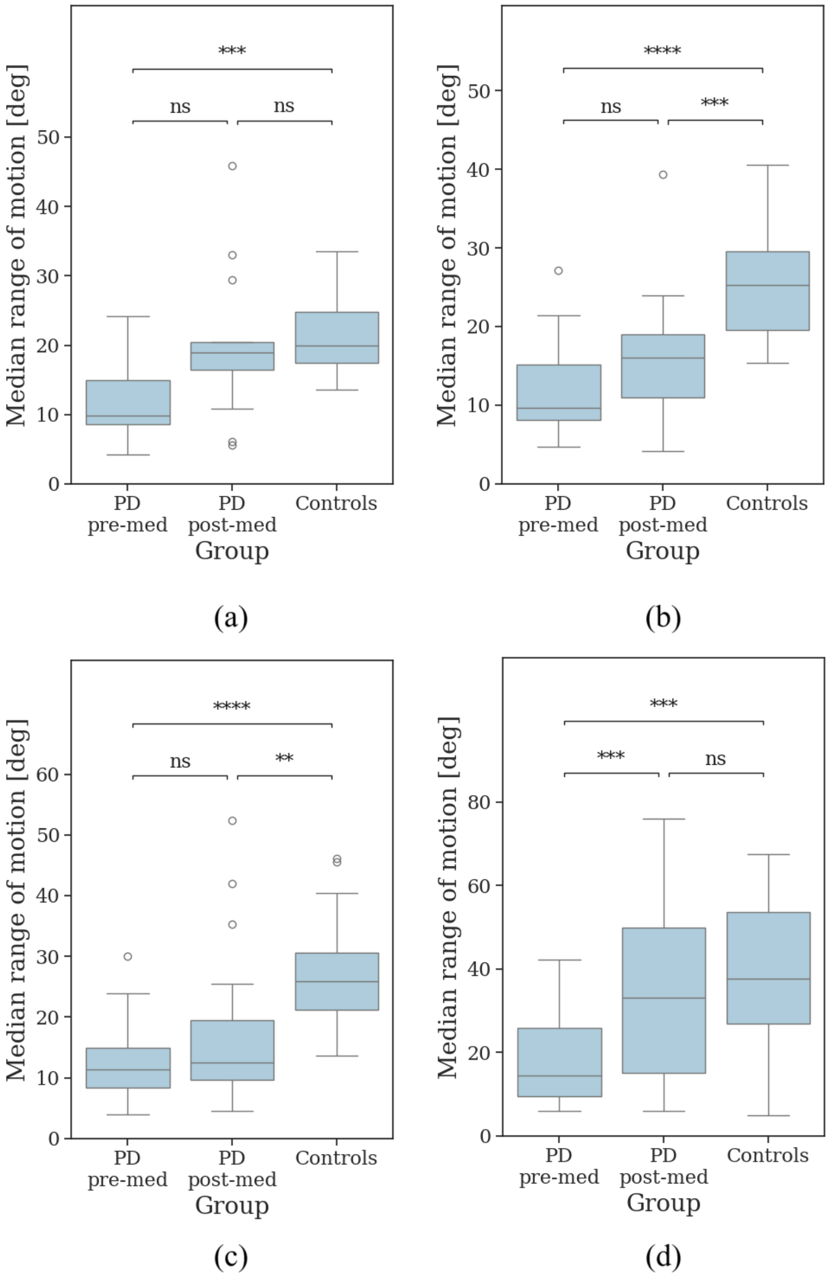


**Fig. S14**: The median range of motion in filtered gait segments for the PD group prior to medication intake (pre-med), the PD group after medication intake (post-med), and the control group. The observations are presented for (a) short gait segments ($< 5$ s), (b) moderately long gait segments (5–10 s), (c) long gait segments (10–20 s), and (d) very long gait segments ($\geq 20$ s).

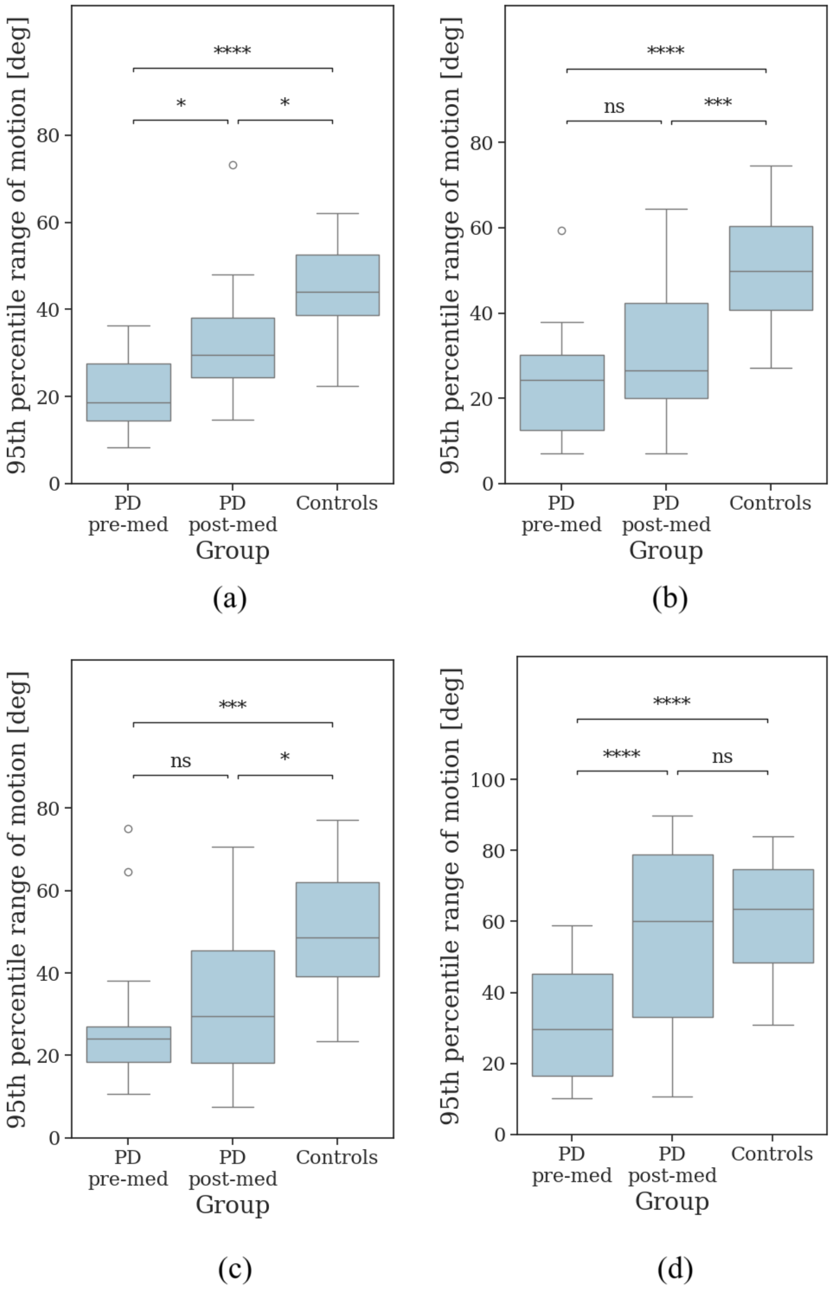


**Fig. S15**: The $95^{\text{th}}$ percentile range of motion in filtered gait segments for the PD group prior to medication intake (pre-med), the PD group after medication intake (post-med), and the control group. The observations are presented for (a) short gait segments (< 5 s), (b) moderately long gait segments (5–10 s), (c) long gait segments (10–20 s), and (d) very long gait segments ($\geq$ 20 s).

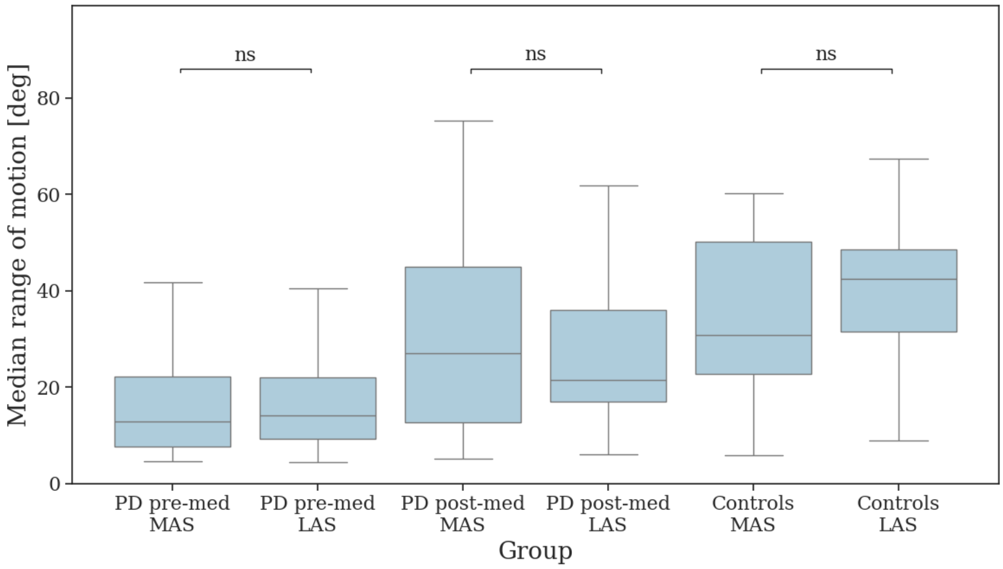


(a)

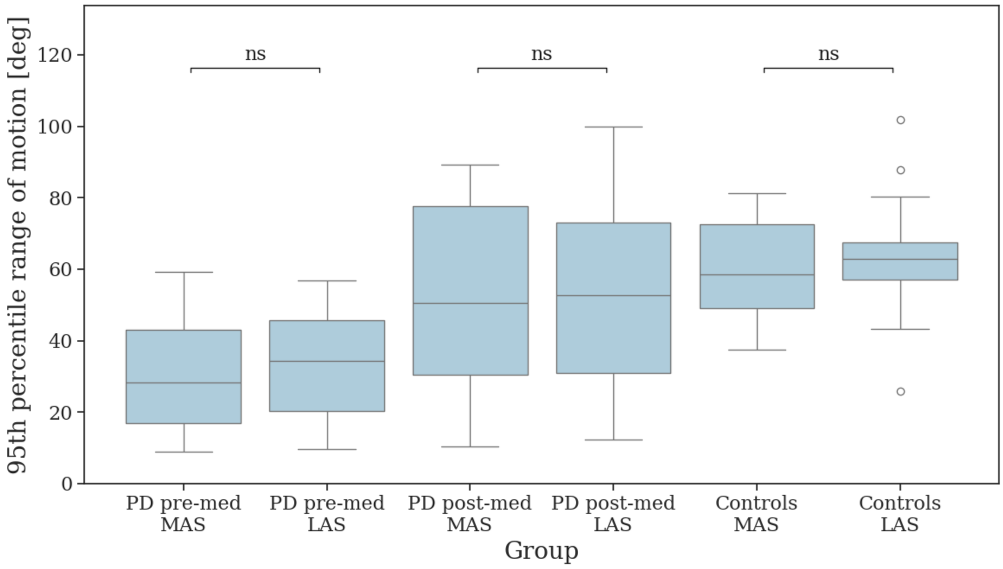


(b)

**Fig. S16**: The (a) median and (b) 95$^{\text{th}}$ percentile range of motion in filtered gait segments, with comparisons between the most affected side (MAS) and least affected side (LAS) of the PD group prior to medication intake (pre-med), the PD group after medication intake (post-med), and the control group.
